# Exploring the interface between adolescent dysmenorrhoea and endometriosis: a protocol for a cohort and nested case–control study within the QResearch Database

**DOI:** 10.1136/bmjopen-2022-069984

**Published:** 2023-02-14

**Authors:** Sharon Dixon, Tom A Ranger, Judith Burchardt, Martina Patone, Andrew JHL Snelling, Katy Vincent, Julia Hippisley-Cox

**Affiliations:** 1 Nuffield Department of Primary Care Health Sciences, University of Oxford, Oxford, UK; 2 Nuffield Department of Women's and Reproductive Health, Oxford University, Oxford, UK

**Keywords:** EPIDEMIOLOGY, GYNAECOLOGY, PRIMARY CARE

## Abstract

**Introduction:**

Dysmenorrhoea affects up to 70%–91% of adolescents who menstruate, with approximately one-third experiencing severe symptoms with impacts on education, work and leisure. Dysmenorrhoea can occur without identifiable pathology, but can indicate underlying conditions, including congenital genital tract anomalies or endometriosis. There is a need for evidence about the management and incidence of dysmenorrhoea in primary care, the impact of treatments in adolescence on long-term outcomes and when to consider the possibility of endometriosis in adolescence.

**Methods and analysis:**

This study aims to improve the evidence base for adolescents presenting to primary care with dysmenorrhoea. It comprises three interlinked studies. Using the QResearch Database, the study population includes all female at birth participants aged 10–19 years any time between 1 January 2000 and 30 June 2021. We will undertake (1) a descriptive study documenting the prevalence of coded dysmenorrhoea in primary care, stratified by demographic variables, reported using descriptive statistics; (2) a prospective open cohort study following an index cohort of all adolescents recorded as attending primary care with dysmenorrhoea and a comparator cohort of five times as many who have not, to determine the HR for a diagnosis of endometriosis, adenomyosis, ongoing menstrual pain or subfertility (considered singly and in combination) anytime during the study period; and (3) a nested case–control study for adolescents diagnosed with endometriosis, using conditional logistic regression, to determine the OR for symptom(s) preceding this diagnosis.

**Ethics and dissemination:**

The project has been independently peer reviewed and received ethics approval from the QResearch Scientific Board (reference OX46 under REC 18/EM/0400).

In addition to publication in peer-reviewed academic journals, we will use the combined findings to generate a resource and infographic to support shared decision-making about dysmenorrhoea in community health settings. Additionally, the findings will be used to inform a subsequent qualitative study, exploring adolescents’ experiences of menstrual pain.

**STRENGTHS AND LIMITATIONS OF THIS STUDY**

- Focusing on the adolescent period will add nuance to existing epidemiological studies using primary care databases exploring the interface between presentation with dysmenorrhoea and diagnosis with endometriosis.
- We will be able to interrogate the findings of this study with a subsequent qualitative study, which will allow exploration and context for questions identified in this research.
- This project is dependent on primary care coding, which may be incomplete. We can explore coded dysmenorrhoea, but this may not represent all presentations with dysmenorrhoea, and some of the variables in our analysis are not linked to structural drivers for recording, such as the Quality and Outcomes Framework.
- Recognition of adolescent endometriosis has likely evolved throughout the study period. We will be able to document and describe this, but it could influence inferences about risk.

## Introduction

Dysmenorrhoea is common among teenagers who menstruate, with prevalence rates of approximately 70%–91%.^1^ Moreover, approximately one-third of these teenagers experience severe dysmenorrhoea,^2^ which is a significant cause of school^3^ and workplace absence,^4^ and is associated with mood disorders and other chronic pain syndromes.^5^ Furthermore, the experience of dysmenorrhoea itself is associated with changes to the central nervous system, including an increased sensitivity to noxious stimuli.^6^ Despite the existence of therapeutic interventions that can help treat pain, community surveys suggest that many young people with period pain do not seek medical help or advice,^7–9^ which highlights the importance of developing resources and services supporting the recognition and treatment of pain.

Dysmenorrhoea can occur in the absence of identifiable underlying pathology (termed primary dysmenorrhoea) or be associated with other conditions, including endometriosis.^10^ Endometriosis occurs in approximately 2%–10% of adult women and is associated with subfertility and chronic pelvic pain.^11–13^ Adolescent endometriosis is believed to be under-recognised^10 14^; however, many women diagnosed with endometriosis in adulthood report symptom onset in adolescence.^14^ Furthermore, there are documented significant delays for adolescents and adults between presentation with symptoms and receiving a diagnosis of endometriosis,^15–18^ and there are concerns that delay in diagnosis contributes to disease progression.^13 14 19^ Therefore, it is critical to understand possible earlier clinical signs and symptoms.

The currently published evidence about the incidence of adolescent endometriosis comes predominantly from tertiary or specialised surgical gynaecological units.^20 21^ There is less knowledge of the prevalence or incidence in the community.^21^ For frontline primary care clinicians, working with this tertiary care estimate risks a denominator problem because the incidence in the community is likely to be significantly lower than in people presenting to specialist clinics. Without knowing what percentage of young people are seen and then referred for onward specialist care for severe dysmenorrhoea, this risk or incidence can be difficult to use, contextualise or communicate in community settings. This is important because the only way to definitively rule out endometriosis is with a laparoscopy,^22^ a procedure associated with risk.^23^

Uncertainties or unknowns in adolescent endometriosis include the lack of long-term outcome data, and whether interventions in teenagers influence long-term outcomes.^21 24^ Alongside the uncertainty about prevalence, there is also uncertainty about whether symptom and menstrual cycle suppression with hormonal treatment might offer potential reductions in the risk of both long-term and short-term outcomes of endometriosis^24 25^ or whether this instead risks masking symptoms, contributes to endometriosis progression or delays diagnosis, potentially leading to an increased risk of adverse outcomes.^18 25 26^

There is evidence that adolescents with endometriosis may present with different symptom patterns from adult women.^27 28^

We are aware of no evidence about the incidence, management or long-term outcomes of teenagers seen in UK primary care with dysmenorrhoea. A recent survey of 442 secondary school students in England reported that 93.6% experienced period pain and 29.5% of those who replied to the survey had seen a doctor about their periods.^29^

In the existing research which has used routinely collected health data to understand patterns of presentation and journeys to diagnosis for endometriosis from UK primary care, findings about adolescents have not been reported separately.^15–17^ In this study, we will use comparable methods with those used to delineate risk and symptomatic features suggesting endometriosis in adult women, but focus specifically on adolescents.

The covariates included in this study are derived from our review of the existing epidemiological literature,^27^ including extrapolation from studies on adult women,^15–17^ and our expert clinical experience, including clinicians working in primary care and adolescent gynaecology. There is very little adolescent-specific (or primary care) evidence or resources to support adolescents with menstrual pain seeking advice and treatment.^30^ There is uncertainty about who to refer, or when, and there is a need for evidence about the likelihood of associated conditions, long term sequelae, or ongoing pain, that is, the natural history of adolescent dysmenorrhoea.^24 31^ These are the questions which this research seeks to address.

### Research aims

The overarching research aim is to improve the evidence base underpinning the diagnosis and care for teenagers with dysmenorrhoea. This study addresses three related but separate research questions, which interface to inform the central research aim:

- What is the prevalence of recorded dysmenorrhoea (or period pain) in adolescents accessing primary care?
- What is the risk of a diagnosis of endometriosis for women with dysmenorrhoea in adolescence recorded in their primary care record?
- What are primary care relevant risk factors for a diagnosis of endometriosis in adolescence?

**Figure 1:**
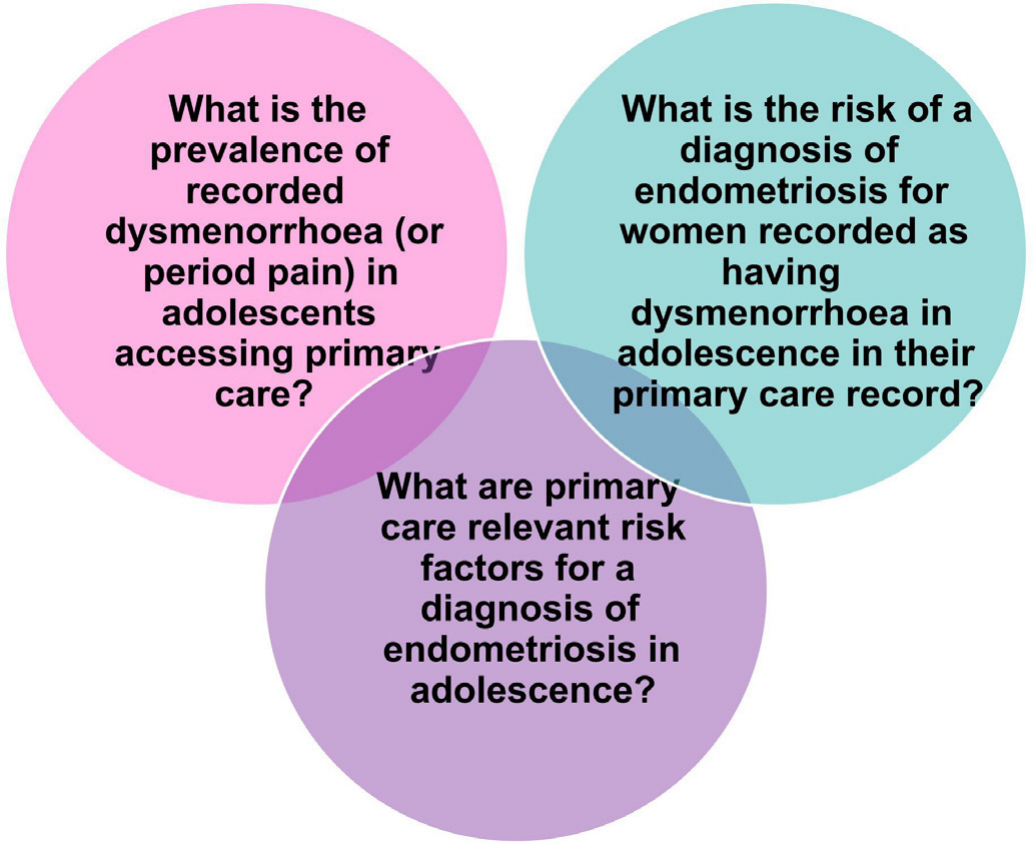
Schematic representation of the research aim and questions.

The research objectives for these three interlinked epidemiological studies are represented schematically in figure 1.

## Methods and analysis

### Patient and public involvement and engagement

This project has been developed with patient and public involvement (PPI) guidance from teenagers and women with lived experience of dysmenorrhoea, including those with and without a diagnosis of endometriosis. Understanding how teenagers with menstrual difficulties can best be supported in primary care emerged as an important research and service need from our previous qualitative research with GPs which included reporting PPI feedback on this work from women with lived experience of endometriosis and menstrual pain.^32^ Questions included when to consider endometriosis, when to refer for investigations and whether symptom suppression with hormonal contraception represented adequate care.

The PPI advisers for this study include adolescents and young adults with lived experience of menstrual pain and adults with experience of pain in adolescence. They helped identify the questions which this study seeks to address, including the focus on presentation with symptoms, the interface between symptoms and possible diagnosis of endometriosis, knowing more about the possible long-term outcomes of adolescent dysmenorrhoea and whether treatment with hormonal contraception helps (or not).

They valued the approach of both moving prospectively from symptoms to diagnosis, alongside looking retrospectively from diagnosis to symptoms. They were interested in exploring the demographic stratification in project A, to consider whether there are any patterns that might suggest unmet needs.

Throughout this study, we will regularly meet with our PPI advisers, and seek their guidance and insights in helping us make sense of our findings, whether they suggest emerging questions, and consider how we can best share and use our results to improve knowledge and care.

### Study design

This study comprises three interlinked population-based projects (designated A, B, C) conducted within the QResearch Database^332^: project A is a descriptive study, project B is a prospective open cohort study and project C is a retrospective nested matched case–control study.

The project components are described individually in turn below and are summarised in table 1.

**Table 1:** Summary table describing the three study epidemiology components

Project	Project research question(s)	Study design	Population included	Comparators/control	Exposure	Primary outcome(s)	Secondary outcome(s)
A	What is the prevalence of recorded dysmenorrhoea (or period pain) in adolescents accessing primary care?	Descriptive study	Female at birth individuals aged 10–19 in any year during 1 Jan 2011–30 June 2021 who are registered with eligible QResearch practices contributing data to the QResearch Database	None	Not applicable	A diagnosis of dysmenorrhoea in primary care	To identify whether there are disparities in recorded attendance at primary care with dysmenorrhoea; prevalence of attending primary care with dysmenorrhoea will be stratified by age, region, quintile of Multiple Deprivation Index and ethnicity
B	What is the risk of a diagnosis of endometriosis for women recorded as having dysmenorrhoea in adolescence in their primary care record? What is the risk of subfertility, chronic pain and ongoing dysmenorrhoea for women recorded as having dysmenorrhoea in adolescence in their primary care records? Does prescribed treatment with hormonal therapies affect the risk of a diagnosis of endometriosis following documented adolescent dysmenorrhoea in the primary care record?	Prospective open cohort study	Female at birth individuals aged 10–19 in any year during 1 Jan 2000–30 June 2021 who are registered with eligible QResearch practices contributing data to the QResearch Database	Five times this number of age-matched randomly selected adolescents who are not recorded as having attended primary care with dysmenorrhoea	Dysmenorrhoea documented in the primary care record in adolescence	Diagnosis of endometriosis at any age during the study period, defined either within the primary care or hospital record	Diagnosis of adenomyosis, evidence of ongoing menstrual pain, a diagnosis of pelvic pain or a diagnosis of subfertility. We will investigate these individually and also in combination as composite outcomes. We will document the number of prescriptions (hormonal contraception, GnRH analogues, tranexamic acid, analgesics (NSAIDs, opioids, neuropathic pain medications) and referrals (pelvic USS, to secondary care specialist services). We will document the mean (SD) and median (IQR) for the time interval between first presentation with symptoms and a diagnosis of endometriosis, chronic pelvic pain or subfertility. We will determine whether this is significantly different if hormonal treatment or treatment to suppress menstruation is prescribed.
C	What are primary care relevant risk factors for a diagnosis of endometriosis in adolescence?	Nested retrospective case–control study	Patients with a diagnosis of endometriosis in adolescence from the cohort of female at birth individuals aged 10–19 in any year during 1 Jan 2000–30 June 2021 who are registered with eligible QResearch practices contributing data to the QResearch Database	Each case will be matched by age with five controls with replacement (ie, a patient not diagnosed with endometriosis)	Individual symptoms or pattern of symptoms preceding a diagnosis of endometriosis in adolescence	A diagnosis of endometriosis in adolescence (aged 10–19)	We will also explore the number of treatments prescribed in primary care, categorised as follows: hormonal treatments and contraceptives, prescribed non-steroidal anti-inflammatory medication, analgesics, antidepressants, medication to manage menstrual flow. The number of referrals from primary care to gynaecology, fertility services, pain services, pelvic USS. We will calculate the time from presentation with symptoms to definitive diagnosis with endometriosis and determine whether this is different between those prescribed hormonal medication or contraception (documented categorically by number of prescriptions) during adolescence and those not prescribed hormonal medication during adolescence

GnRH, gonadotropin-releasing hormone; NSAIDs, non-steroidal anti-inflammatory drugs; USS, ultrasound scan.

### Data source and variables

QResearch is a large, validated database of over 35 million anonymised electronic primary care health records dating back to 1989. It is representative of the English general population. Patient data are compiled at an individual level from GP practices through the Egton Medical Information Systems electronic health record system with linkages to the National Health Service Digital Hospital Episode Statistics (HES) platform.^33^

The variables included in this study represent outcomes, exposures, lifestyle factors and potential covariates. These are summarised in table 2. Data extracted from QResearch will be derived using SNOMED-CT code groups. HES data will be derived from the International Classification of Diseases 10th Revision code system (ICD-10) and the Office of Population Censuses and Surveys Classification of Interventions and Procedures version 4 (OPCS4) codes.

**Table 2:** Variables included in this study

Type of variable	Project relevant to	Included variables	Variable source
Demographic variables	A, B, C	Geographical region in England (categorical, 10 regions) Townsend deprivation quintile Ethnicity (categorical; white, Indian, Bangladeshi, Pakistani, Chinese, other Asian, Caribbean, black African, other)	QResearch
	A, B, C	Age (continuous variable)	QResearch
Lifestyle variables	A, B, C	Smoking status (categorical; non-smoker, ex-smoker, light smoker <10/day, moderate smoker 10–19/day, heavy smoker >20/day) Body mass index (continuous)	QResearch
Primary exposure	B	Dysmenorrhoea (primary, secondary, not specified, menstrual pain)	QResearch
	C	The pattern of symptoms recorded in the primary care record prior to a diagnosis of adolescent endometriosis	QResearch
Primary outcome	A	Number of adolescents coded with dysmenorrhoea in primary care	QResearch
	B	Diagnosis of endometriosis at any time within the study period	QResearch and HES
	C	Cases are those diagnosed with endometriosis during adolescence	QResearch
Secondary outcomes	A	Demographic variables above	QResearch
	B	Subfertility and infertility Pelvic pain Adenomyosis Ongoing menstrual pain (all dysmenorrhoea codes as above)	QResearch and HES (subfertility, adenomyosis)
	B	Medication and referrals as detailed below	QResearch
Secondary (other) exposures of interest: medications prescribed in primary care	B, C	Hormonal treatments and contraceptives Combined hormonal contraception Progesterone-only contraceptive pills Intrauterine system prescriptions issued by GP practice or coded within primary care records Subdermal contraception codes GnRH analogues (prescriptions issued by GP practice) Prescribed non-steroidal anti-inflammatory medication Naproxen, ibuprofen, cox-2 inhibitors Mefenamic acid Analgesics Codeine, opioids Pregabalin Gabapentin Antidepressants SSRI Amitryptilline, nortryptilline (due to indication for neuropathic analgesia) Medications to manage menstrual flow Tranexamic acid	QResearch
Referrals made	B, C	Referrals from primary care to: Gynaecology Fertility services Pain services Pelvic ultrasound scan Within secondary care: Pelvic MRI and pelvic ultrasound scan Fertility treatment Diagnostic laparoscopy	QResearch OPCS ICD-10
	Potential confounders or covariates		
Gynaecological variables	B, C	Dyspareunia (superficial, deep, not otherwise specified) Post-coital bleeding Polycystic ovarian syndrome Menorrhagia and heavy menstrual bleeding Dysfunctional uterine bleeding Irregular menstrual cycles (irregular, infrequent or frequent bleeding) PID Ovarian cysts Congenital uterine or genital tract anomalies (developmental disorders of the female genital tract) Genital pain Vaginismus Amenorrhoea Premenstrual symptoms Vaginal discharge Ovulation pain Menstrual disorder, not otherwise specified	QResearch and HES (for ovarian cysts, PID, congenital uterine or genital tract anomalies)
Non-gynaecological variables	B, C	Depression Anxiety Irritable bowel syndrome Diarrhoea Constipation Nausea and vomiting dysuria Cystitis and UTI (chronic, recurrent, simple) Rectal or anal pain Dyschezia (difficult or painful defecation) Rectal bleeding Migraine Low back pain Fatigue Bloating Fibromyalgia	QResearch
Pregnancy-related outcomes	B	Pregnancy Live birth Ectopic pregnancy	QResearch and HES

GnRH, gonadotropin-releasing hormone; GP, general practitioner; HES, Hospital Episode Statistics; ICD-10, International Classification of Diseases 10th Revision; OPCS, Office of Population Censuses and Surveys Classification of Interventions and Procedures; PID, pelvic inflammatory disease; SSRI, selective serotonin reuptake inhibitor; UTI, urinary tract infection.

The variables selected for inclusion in this study have been derived from published literature about symptoms that might suggest endometriosis including studies which have used primary care databases to determine symptom patterns^15–17 26^, and evidence about symptoms in adolescence.^5 27 28^ These include gynaecological symptoms, as well as symptoms from the gastrointestinal tract,^18^ the urinary tract and the musculoskeletal system.^10 13 15 17 24 27 28^

We have not included variables which may be relevant risk factors for endometriosis, but where coding in primary care is likely to be incomplete or unreliable. Following expert guidance, we have not included age at menarche, family history of menstrual disorders or anovulatory cycles (where primary care would not usually be able to ascertain or validate the code). However, we have included irregular cycles which may partially reflect anovulation, but which is a descriptive code and so can be meaningfully recorded within primary care records.

The code groups were developed within QWeb using SNOMED-CT, with input for our code group definitions from the published literature and expert consultation.

The SNOMED, ICD-10, drug group and OPCS codes contributing data to each of the variable code groups are available at https://www.qresearch.org/data/qcode-group-library/.

Demographic and lifestyle variables will be extracted from the primary care records. Data related to the exposure, and primary and secondary outcomes will be extracted from the GP records, with hospital-linked data additionally used for the primary and secondary diagnostic outcomes and referrals as detailed below.

### Study period

For the cohort and case–control studies (B, C), the study period is from 1 January 2000 to 30 June 2021, the most recent date for which complete data are expected. For study A, the study period is from 1 January 2011 to 30 June 2021.

### Inclusion criterion

We will use the WHO definition of adolescence, which is 10–19 years old.^34^

For all components of the study, the inclusion criteria are female at birth individuals aged 10–19 years in any year during the study period and who are registered with practices contributing data to the QResearch Database. Additional inclusion rules for the different study components are described within individual project method sections.

### Exclusion criterion

Individuals who are not female at birth or not aged 10–19 years during the study period.

### Method and statistics plan: project A

**Research question**: What is the prevalence of recorded dysmenorrhoea in adolescents accessing primary care?

**Study design**: descriptive study.

**Inclusion**: individuals aged 10–19 years in any year between 1 January 2011 and 30 June 2021.

**Exclusion criterion**: no additional rules.

**Outcomes**: a primary care diagnosis of dysmenorrhoea.

**Exposure**: not applicable.

**Confounding variables:** outcomes will be stratified by the following demographic variables: age, region, quintile of Multiple Deprivation Index and ethnicity.

**Statistical methods:** the prevalence of adolescent dysmenorrhoea in primary care will be reported by year of diagnosis and stratified by age, ethnicity, quintile of deprivation and region.

### Method and statistics plan: project B

**Research questions:**

- What is the risk of a diagnosis of endometriosis for women with dysmenorrhoea in adolescence recorded in their primary care record?
- What is the risk of subfertility, chronic pain and ongoing dysmenorrhoea for women recorded as having dysmenorrhoea in adolescence in their primary care records?
- Does prescribed treatment with hormonal therapies affect the risk of a diagnosis of endometriosis following documented adolescent dysmenorrhoea in the primary care record?

**Study design:** prospective open cohort study.

**Inclusion criterion:** all adolescents who are recorded as attending primary care with dysmenorrhoea and a comparator cohort comprising five times this number of age-matched, randomly selected adolescents without replacement who are not recorded as having attended primary care with dysmenorrhoea.

**Exclusion criterion**: no additional rules.

**Primary outcomes:** diagnosis of endometriosis at any age during the study period, defined either within the primary care or hospital record.

**Secondary outcomes:** diagnosis of adenomyosis, evidence of ongoing menstrual pain, a diagnosis of pelvic pain or a diagnosis of subfertility. We will investigate these individually and also in combination as composite outcomes.

**Exposure:** dysmenorrhoea documented in the primary care record in adolescence.

**Potential covariates or confounding variables to be considered:**

- Gynaecological variables: dyspareunia (superficial, deep, not otherwise specified), post-coital bleeding, polycystic ovarian syndrome (PCOS), menorrhagia and heavy menstrual bleeding, dysfunctional uterine bleeding, irregular menstrual cycles (infrequent and frequent), pelvic inflammatory disease, ovarian cysts, congenital uterine or genital tract anomalies (developmental disorders of the female genital tract).
- Non-gynaecological variables: depression, anxiety, irritable bowel syndrome, diarrhoea, constipation, nausea, irritable bladder, dysuria, chronic cystitis, cystitis and urinary tract infection (UTI), rectal or anal pain, dyschezia, rectal bleeding, migraine, low back pain, fatigue, bloating, fibromyalgia.
- Pregnancy-related variables: pregnancy, live birth, ectopic pregnancy.
- Demographic and lifestyle variables: geographical region in England (categorical, 10 regions), Townsend deprivation quintile, ethnicity, age, body mass index (BMI), smoking.
- Medication related: hormonal treatments and contraceptives, prescribed non-steroidal anti-inflammatory drug (NSAID) medication, analgesics, antidepressants, medication to manage menstrual flow (table 2).
- Referrals from primary care to gynaecology, fertility services, pain services, pelvic ultrasound scan.

**Statistical methods:** HRs and 95% CIs for each outcome will be estimated using Cox regression with adjustment for prespecified covariates in multivariable models. The proportional hazards assumption will be checked visually using log minus log survival plots for each regression model. Effect estimates for each symptom will be calculated and visualised in forest plots. Because subjects may enter the cohort at different times or may not yet have experienced one or more of the endpoints, we will use a log-rank test to compare time to event curves. We will run separate models for each primary and composite outcome.

The time interval between documentation of symptoms in the primary care record and referral to secondary care or receiving a diagnosis of endometriosis will be mapped using Kaplan-Meier plots.

Potential confounders will be accounted for in the Cox model. We will include consideration of other menstrual diagnoses and symptoms, contraceptive use and pelvic inflammatory disease as potential confounders. In light of the exploratory nature of this project, we have included a wide range of potentially relevant covariates, including gynaecological and non-gynaecological variables. We will document descriptive statistics (frequency and mean/SD or median/IQR) for the number of occurrences of the following variables as detailed in table 2: hormonal treatments and contraceptives, prescribed non-steroidal anti-inflammatory medication, analgesics, antidepressants, medication to manage menstrual flow, referrals from primary care to gynaecology, fertility services, pain services and pelvic ultrasound scan.

We will document the mean (SD) and median (IQR) for the time interval between first presentation with symptoms and a diagnosis of endometriosis, chronic pelvic pain or subfertility. We will determine whether this is significantly different if hormonal treatment or treatment to suppress menstruation is prescribed.

### Method and statistics plan: project C

**Research question**: What are primary care relevant risk factors for a diagnosis of endometriosis in adolescence?

**Research design:** retrospective nested matched case–control study.

**Inclusion criterion:** patients with a diagnosis of endometriosis in adolescence. Each case will be matched by age with five controls with replacement (ie, a patient not diagnosed with endometriosis).

**Exclusion criterion**: no additional rules.

**Outcome/case definition**: diagnosis of endometriosis in adolescence.

**Exposure:** individual symptoms or pattern of symptoms preceding a diagnosis of endometriosis in adolescence.

**Potential confounders or covariates:**

- Gynaecological variables: dyspareunia (superficial, deep, not otherwise specified), post-coital bleeding, PCOS, menorrhagia and heavy menstrual bleeding, dysfunctional uterine bleeding, irregular menstrual cycles, pelvic inflammatory disease, ovarian cysts, congenital uterine or genital tract anomalies (developmental disorders of the female genital tract).
- Non-gynaecological variables: depression, anxiety, irritable bowel syndrome, diarrhoea, constipation, nausea, irritable bladder, dysuria, bladder pain syndrome/interstitial cystitis, cystitis and UTI, rectal or anal pain, dyschezia, rectal bleeding, migraine, low back pain, fatigue, bloating, fibromyalgia.
- Demographic and lifestyle variables: geographical region in England (categorical, 10 regions), Townsend deprivation quintile, ethnicity, age, BMI, smoking.
- Medication related: hormonal treatments and contraceptives, prescribed NSAID medication, analgesics, antidepressants, medication to manage menstrual flow (table 2).
- Referrals from primary care to gynaecology, fertility services, pain services, pelvic ultrasound scan.

**Statistics plan:** using conditional logistic regression, we will calculate unadjusted and adjusted ORs for adolescent endometriosis for identified exposures. We will consider whether there are exposures that alter the likelihood of a diagnosis of adolescent endometriosis. Symptoms with an OR >1.2 for risk of endometriosis with associated 95% CI which does not contain the value 1 will be considered clinically relevant.

We will explore the number of treatments prescribed in primary care, categorised as follows: hormonal treatments and contraceptives, prescribed non-steroidal anti-inflammatory medication, analgesics, antidepressants, medication to manage menstrual flow, the number of referrals from primary care to gynaecology, fertility services, pain services and pelvic ultrasound scan.

We will calculate the time from presentation with symptoms to definitive diagnosis with endometriosis and determine whether this is different between those prescribed hormonal medication or contraception (documented categorically by number of prescriptions) during adolescence and those not prescribed hormonal medication during adolescence.

#### Sample size calculation

We have not been able to identify any published evidence about the incidence or prevalence of coded dysmenorrhoea in adolescence in primary care in the UK or worldwide. Therefore, project A will generate information which will help us refine our calculations of feasibility and necessary parameters for the subsequent studies. Recognising the lack of established recording prevalence, we will include every case within the QResearch Database to optimise the likelihood of attaining statistical power.

In recognition of this uncertainty, we have adopted a conservative approach to our sample size calculation and have conducted a formal statistical power and feasibility calculation.

For project B, estimating a conservative HR of 1.2 for developing one of the primary endpoints, with significance of 0.05 and power of 0.8, we estimate that we would need at least 945 patients with each diagnosis recorded and a comparator cohort of at least 15 742 controls. We consider this to be feasible, given the size of the database.

For project C, we calculate that we would need 2873 cases with an exposure probability in the control group of 0.1 (or 5376 cases for an exposure probability of 0.05) to estimate an OR of 1.2 with significance of 0.05 and power of 0.8, considering a matching of 1:5. We consider this to be feasible based on the number of adolescents documented with endometriosis in previous UK-based population cohort studies.

#### Plan for missing data

We anticipate missing data for ethnicity, BMI and smoking status. We will assume that these data are missing at random (MAR).

Missing values will be imputed using multiple imputation with chained equations using all outcome and exposure variables in the model. Five imputations will be carried out and Rubin’s rules will be used to pool model estimates across the imputed datasets.

### Sensitivity analysis

#### Complete versus imputed dataset

We will do a complete case analysis as sensitivity analysis to check the MAR assumption. Results from both multiple imputation and complete case analyses will be reported.

#### Estimation of the rate ratio of endometriosis diagnosis in adolescence in those with and without documented coding of dysmenorrhoea

Case–control study C explores cases of endometriosis, with controls with and without dysmenorrhoea. We will also conduct a sensitivity analysis within the cohort coded with dysmenorrhoea with cases being those with dysmenorrhoea and adolescent endometriosis, and controls with dysmenorrhoea without diagnosed adolescent endometriosis, to identify other relevant patterns or variables that may affect or predict risk.

### Ethics

The project has been independently peer reviewed and received ethics approval from the QResearch Scientific Board (reference OX46 under REC 18/EM/0400).^35^

### Dissemination

Our findings will be reviewed with our PPI advisers, who will advise on dissemination and communication approaches. The findings of these studies will be published in peer-reviewed academic journals and presented at conferences, including seeking cross-disciplinary avenues for research dissemination. We will work with collaborating advocacy organisations and patient groups to seek opportunities to share what we have learnt and consider opportunities for how this can be used to improve care.

We will use the combined findings to generate an evidence-based infographic to support shared decision-making about dysmenorrhoea in community care settings.

Finally, these projects will inform a subsequent qualitative study, exploring adolescents’ experiences of menstrual pain. With their input, we will write accessible summaries about important aspects of painful periods which will be freely available on a widely used health experience website (Healthtalk.org).

## Discussion

This study explores the interface between a commonly experienced symptom in adolescence and the likelihood of subsequent diagnoses including endometriosis. By doing this, we seek to identify factors that will help clinicians and patients navigate their care journeys which will likely begin in primary care with presentation with as yet unexplained symptoms. These epidemiological studies will inform a subsequent qualitative study exploring young people’s experiences of menstrual pain, including those who have (or have not) sought care, and who do (or do not) have a diagnosis of endometriosis.

The paucity of evidence in this field has led us to propose an initial comparison of cases (all adolescents coded with dysmenorrhoea) against controls without coded dysmenorrhoea. Dysmenorrhoea is common, affecting approximately 70%–91% of the adolescent population. It is not known who, how, and when individuals present to primary care and we do not know how complete recording or coding is. Therefore, it is likely that some of our control group will also experience dysmenorrhoea.

For young people deciding whether to access care, educators, families and frontline gatekeeper clinicians, an important initial question is ‘how likely is it that any young person with period pain has an underlying condition?’, ‘What can I advise about long-term outcomes?’ and ‘What can we advise about treatments now?’ By including all adolescents who either did or did not present with dysmenorrhoea when we explore the likelihood of a diagnosis of endometriosis means that if there are systemic or structural reasons that affect attendance for care, then we may be able to consider these.

However, knowing how the risk of endometriosis can be stratified among adolescents who do present to primary care with dysmenorrhoea is also important. Recognising this, we plan to undertake a sensitivity analysis contrasting rates of endometriosis in adolescents with documented dysmenorrhoea within study C, to see whether there are factors that help identify which adolescents with dysmenorrhoea may be more likely to have endometriosis.

Understanding more about which adolescents with dysmenorrhoea to refer for further investigations is critical to improving care journeys. Knowing more about the impacts of treatment with hormonal medications in adolescence is also crucial to inform guidance for adolescents, their families and health professionals who look after them.

The aims of this project align with health policy within the Women’s Health Strategy which reinforces the need for awareness and resources to support menstrual well-being and care for those with endometriosis.^36^

This approach will bridge and add to existing literature, which does not focus on the primary care interface crucial to accessing care in the UK health system.

A limitation of this project is its reliance on primary care coding, which may be incomplete or inaccurate. Data entry and recording in primary care are conducted to support clinical care and not predominantly to support research. However, existing understanding of endometriosis symptomatology in primary care is derived using comparable methods, although adolescents are not considered or reported separately.^15–17^ Therefore, there is methodological justification for using this approach to consider adolescents as proposed in this study.

Limitations include the possibility of bias due to missing data, or from data misclassification, for example, miscoding of symptoms or diagnoses. Adolescents with dysmenorrhoea may not have this documented in their primary care record, either because they have not attended for care, not reported this (or been heard) or not had this recorded as a SNOMED code (for example, if it is documented within consultation free text).

With menstrual symptoms, social acceptability bias may exist and may not be uniformly experienced across the population. The stratified analysis in project A may highlight areas for exploration in the aligned qualitative study. Recorder and recall bias are potentially relevant, whereby symptoms are better coded when a referral or treatment is planned, which will not apply to the comparator group.

Smoking and BMI are more likely to be recorded for young people prescribed hormonal treatment as these prescriptions represent a template-driven opportunity to record these data. These data are more likely to be complete for young people prescribed hormonal treatment in primary care (and would not capture these data for those whose hormonal treatment was prescribed in other healthcare settings, for example, community sexual health clinics). Furthermore, the adolescents using sexual health clinics (rather than primary care) may systematically differ from each other (for example, if they vary by location such as rural settings). Patterns of contraception use may also change over time, as demonstrated by the ‘pill scare’ of 1995 which caused a reduction in contraceptive pill use.^37^ Menstruation and endometriosis do not link with any Quality and Outcomes Framework codes, although long-acting contraception has done,^38^ which may act as a driver towards contraception being coded rather than pain or symptoms. Adolescence to adulthood can be a time of mobility, for example, if adolescents leave home to travel elsewhere for work or study, which might limit the duration of follow-up if people de-register from their GP when they move. Furthermore, these changes may not be uniformly distributed across social or demographic factors.

Finally, adolescent gynaecological units are not uniformly distributed across the country, and access to specialist adolescent services, tertiary or super-specialist care may influence referral rates and patterns of identification and referral.^39^ If identified through stratification against region, this could offer a valuable finding.

We recognise that these represent a significant limitation of this approach. We will perform sensitivity analyses to compare robustness of results between complete case and imputed analyses.

However, this project proposes a novel approach to develop understanding of a complex and important challenge when moving from symptoms of menstrual pain in adolescence to diagnosis of endometriosis, which is an under-researched and under-recognised health condition, which can have significant adverse impacts on women’s health and well-being.

## Supplementary Material

Reviewer comments

Author's manuscript
