# Supplementary material for: Exploring the interface between adolescent dysmenorrhoea and endometriosis: a protocol for a cohort and nested case–control study within the QResearch Database
Source: BMJ Open. 2023 Feb 14;13(2):e069984. doi: 10.1136/bmjopen-2022-069984 (PMC9930556; doi:10.1136/bmjopen-2022-069984)
Supplement: Author's manuscript [file bmjopen-2022-069984.draft_revisions.pdf]

BMJ Open is committed to open peer review. As part of this commitment we make the peer review history of every article we publish publicly available.

When an article is published we post the peer reviewers' comments and the authors' responses online. We also post the versions of the paper that were used during peer review. These are the versions that the peer review comments apply to.

The versions of the paper that follow are the versions that were submitted during the peer review process. They are not the versions of record or the final published versions. They should not be cited or distributed as the published version of this manuscript.

BMJ Open is an open access journal and the full, final, typeset and author-corrected version of record of the manuscript is available on our site with no access controls, subscription charges or pay-per-view fees (http://bmjopen.bmj.com).

If you have any questions on BMJ Open's open peer review process please email info.bmjopen@bmj.com

BMJ Open

Exploring the interface between adolescent dysmenorrhoea and endometriosis: A protocol for a cohort and nested case-control study within the QResearch Database

Journal:	*BMJ Open*
Manuscript ID	bmjopen-2022-069984
Article Type:	Protocol
Date Submitted by the Author:	15-Nov-2022
Complete List of Authors:	Dixon, Sharon; University of Oxford, Nuffield Department of Primary Care Health Sciences<br>Ranger, Tom; Oxford University, Primary Care Health Sciences<br>Burchardt, Judith; Oxford University, Primary Care Health Sciences<br>Patone , Martina ; University of Oxford,<br>Snelling, Andrew; Oxford University, Nuffield Department of Primary Care Health Sciences<br>Vincent, Katy; Oxford University, Nuffield Department of Women's and Reproductive Health<br>Hippisley-Cox, Julia; University of Oxford, Nuffield Department of Primary Care Sciences
Keywords:	EPIDEMIOLOGY, GYNAECOLOGY, PRIMARY CARE

SCHOLARONE™
Manuscripts

Exploring the interface between adolescent dysmenorrhoea and endometriosis: A protocol for a cohort and nested case-control study within the QResearch Database

*Sharon Dixon[1], Tom Ranger[1], Judith Burchardt[1], Martina Patone[1], Andrew Snelling[1], Katy Vincent[2], Julia Hippisley-Cox[1]

1= Nuffield Department of Primary Care Health Sciences, University of Oxford

2=Nuffield Department of Women's and Reproductive Healthcare, University of Oxford.

Corresponding author:

Sharon Dixon

Radcliffe Primary Care Building,

Radcliffe Observatory Quarter,

Woodstock Road, Oxford. OX2 6GG.

Tel: +44 1865 617855

Funding source:

This study was funded as part of Sharon Dixon's NIHR Doctoral research fellowship NIHR301787. There is no other relevant funding to declare.

Disclaimer:

The views expressed are those of the author(s) and not necessarily those of the NIHR or the Department of Health and Social Care.

COI:

KV has received research funding from Bayer Healthcare and honoraria for talks and consultancy fees from Bayer Healthcare, AbbVie and Eli Lilly. The remaining authors declare no relevant COI

Word count: 4043

Number of tables: 2

Number of figures: 1

Number of references: 39

Sharon Dixon sharon.dixon@phc.ox.ac.uk

For peer review only

Abstract:

Introduction:

Dysmenorrhoea affects approximately 70-91% of adolescents who menstruate, with approximately one third experiencing severe symptoms with impacts on education, work and leisure. Dysmenorrhoea can occur without identifiable pathology, but can indicate underlying conditions, including congenital genital tract anomalies or endometriosis. There is a need for evidence about the management and incidence of dysmenorrhoea in primary care, the impact of treatments in adolescence on long-term outcomes, and when to consider the possibility of endometriosis in adolescence.

Methods and analysis:

This study aims to improve the evidence base for adolescents presenting to primary care with dysmenorrhoea. It comprises three inter-linked studies. Utilising the QResearch database, the study population includes all female at birth participants aged 10-19 any time between 1.1.2000 and 30.6.2021. We will undertake (i) a descriptive study documenting the prevalence of coded dysmenorrhoea in primary care, stratified by demographic variables, reported using descriptive statistics; (ii) a prospective open cohort study following an index cohort of all adolescents recorded as attending primary care with dysmenorrhoea and a comparator cohort of five times as many who have not, to determine the hazard ratio for a diagnosis of endometriosis, adenomyosis, ongoing menstrual pain or sub-fertility (considered singly and in combination) anytime during the study period; and (iii) a nested case-control study for adolescents diagnosed with endometriosis, using conditional logistic regression, to determine the odds ratio for symptom(s) preceding this diagnosis.

Ethics and Dissemination:

The project has been independently peer-reviewed and received ethics approval from the QResearch Scientific Board (reference OX46 under REC 18/EM/0400).

In addition to publication in peer reviewed academic journals, we will utilise the combined findings to generate a resource and infographic to support shared decision-making about dysmenorrhoea in community health settings. Additionally, the findings will be used to inform a subsequent qualitative study, exploring adolescent's experiences of menstrual pain.

Strengths and limitations:

- This study will explore improvements in the diagnosis of endometriosis, focussing on adolescents, which is an identified service and research priority.
- By exploring the relationship between adolescent dysmenorrhoea and endometriosis both prospectively, starting from symptoms then diagnosis, and retrospectively, starting with the diagnosis and looking back at symptoms, this study takes a novel approach of interrogating the central research question using three separate but inter-linked projects which will add rigor and depth to the findings.
- We will be able to interrogate the findings of this study with a subsequent qualitative study, which will allow exploration and context for questions identified in this research.
- This project is dependent on primary care coding, which may be incomplete. We can explore coded dysmenorrhoea, but this may not represent all presentations with dysmenorrhoea, and some of the variables in our analysis are not linked to structural drivers for recording, such as the QOF framework.
- Recognition of adolescent endometriosis has likely evolved throughout the study period. We will be able to document and describe this, but it could influence inferences about risk.

For peer review only

Introduction:

Dysmenorrhoea is common amongst teenagers who menstruate, with prevalence rates of approximately 70- 91%[1]. Moreover, approximately a third of these teenagers experience severe dysmenorrhoea[2], which is a significant cause of school[3] and workplace absence,[4] and is associated with mood disorders and other chronic pain syndromes[5]. Furthermore, the experience of dysmenorrhoea itself is associated with changes to the central nervous system, including an increased sensitivity to noxious stimuli[6]. Despite the existence of therapeutic interventions that can help treat pain, community surveys suggest that many young people with period pain do not seek medical help or advice[7-9], which highlights the importance of developing resources and services supporting the recognition and treatment of pain.

Dysmenorrhoea can occur in the absence of identifiable underlying pathology (termed primary dysmenorrhoea) or be associated with other conditions, including endometriosis[10]. Endometriosis occurs in approximately 2-10% of adult women and is associated with sub-fertility and chronic pelvic pain[11,12]. Adolescent endometriosis is believed to be under-recognised[10,14], however many women diagnosed with endometriosis in adulthood report symptom onset in adolescence[14]. Furthermore, there are documented significant delays for adolescents and adults between presentation with symptoms and receiving a diagnosis of endometriosis[15-18] and there are concerns that delay in diagnosis contributes to disease progression[14,19]. Therefore it is critical to understand possible earlier clinical signs and symptoms.

The currently published evidence about the incidence of adolescent endometriosis comes predominantly from tertiary or specialised surgical gynaecological units. [20,21]. There is less knowledge of the prevalence or incidence in the community[21]. For front line primary care clinicians working with this tertiary care estimate risks a denominator problem because the incidence in the community is likely to be significantly lower than in people presenting to specialist clinics. Without knowing what percentage of young people are seen and then referred for onward specialist care for severe dysmenorrhoea, this risk or incidence can be difficult to utilise, contextualise, or communicate in community settings. This is important because the only way to definitively rule out endometriosis is with a laparoscopy[22], a procedure associated with risk[23].

Uncertainties or unknowns in adolescent endometriosis include the lack of long-term outcome data, and whether interventions in teenagers influence long-term outcomes[21,24]. Alongside the uncertainty about prevalence, there is also uncertainty about whether symptom and menstrual cycle suppression with hormonal treatment might offer potential reductions in the risk of both long- and short-term outcomes of endometriosis [24,25] or whether this instead risks masking symptoms, contributes to endometriosis progression, or delays diagnosis, potentially leading to an increased risk of adverse outcomes[18,25,26] .

There is evidence that adolescents with endometriosis may present with different symptom patterns from adult women[27,28].

We are aware of no evidence about the incidence, management, or long-term outcomes of teenagers seen in UK primary care with dysmenorrhoea. A recent survey of 442 secondary school students in England reported that 93.6% experienced period pain and 30% of those who replied to the survey had seen their GP[29].

In the existing research which has used routinely collected health data to understand patterns of presentation and journeys to diagnosis for endometriosis from UK primary care, findings about adolescents have not been reported separately[15-17]. In this study, we will use comparable methods to those utilised to delineate risk and symptomatic features suggesting endometriosis in adult women, but focus specifically on adolescents.

The co-variates included in this study are derived from our review of the existing epidemiological literature[27], including extrapolation from studies on adult women[15-17], and our expert clinical experience, including clinicians working in primary care and adolescent gynaecology. There is very little adolescent specific (or primary care) evidence or resources to support adolescents with menstrual pain seeking advice and treatment[30]. There is uncertainty about who to refer, or when, and there is a need for evidence about the likelihood of associated conditions or on-going pain i.e. the natural history of adolescent dysmenorrhoea[31.] These are the questions which this research seeks to address.

For peer review only

Research aims:

The over-arching research aim is to improve the evidence base underpinning the diagnosis and care for teenagers with dysmenorrhoea. This study addresses three related but separate research questions, which interface to inform the central research aim:

A: What is the prevalence of recorded dysmenorrhoea (or period pain) in adolescents accessing primary care?

B: What is the risk of a diagnosis of endometriosis for women with dysmenorrhoea in adolescence recorded in their primary care record?

C: What are primary care relevant risk factors for a diagnosis of endometriosis in adolescence?

The research objectives for these three inter-linked epidemiologic studies are represented schematically in figure 1.

Figure 1: Schematic representation of the research aim and questions.

Methods and analysis:

PPI:

This project has been developed with PPI guidance from teenagers and women with lived experience of dysmenorrhoea, including those with and without a diagnosis of endometriosis. Understanding how teenagers with menstrual difficulties can best be supported in primary care emerged as an important research and service need from our previous qualitative research with GPs which included reporting PPI feedback on this work from women with lived experience of endometriosis and menstrual pain[34]. Questions included when to consider endometriosis, when to refer for investigations, and whether symptom suppression with hormonal contraception represented adequate care.

The PPI advisers for this study include adolescents and young adults with lived experience of menstrual pain and adults with experience of pain in adolescence, and input from endometriosis UK. They helped identify the questions which this study seeks to address, including the focus on presentation with symptoms, the interface between symptoms and possible diagnosis of endometriosis, knowing more about the possible long-term outcomes of adolescent dysmenorrhoea, and whether treatment with hormonal contraception helps (or not).

They valued the approach of both moving prospectively from symptoms to diagnosis, alongside looking retrospectively from diagnosis to symptoms. They were interested in exploring the demographic stratification in project A, to consider whether there are any patterns that might suggest unmet needs.

Throughout this study, we will regularly meet with our PPI advisers, and seek their guidance and insights in helping us make sense of our findings, whether they suggest emerging questions, and consider how we can best share and utilise our results to improve knowledge and care.

Study design:

This study comprises three inter-linked population-based projects (designated A, B, C) conducted within the QResearch database[32]: project A is a descriptive study, project B is a prospective open cohort study and project C is a retrospective nested matched case-control study.

The project components are described individually in turn below and are summarised in Table 1.

For peer review only

Table1: Summary table describing the three study epidemiology components:

Project	Project research question(s)	Study design	Population included	Comparators /control	Exposure	Primary outcome(s)	Secondary Outcome(s)
**A**	What is the prevalence of recorded dysmenorrhoea (or period pain) in adolescents accessing primary care?	Descriptive study	Female at birth individuals aged 10-19 in any year during 1.1.2011 to 30.6.2021 who are registered with eligible QResearch practices contributing data to the QResearch database	None	Not applicable	A diagnosis of dysmenorrhoea in primary care.	To identify whether there are disparities in recorded attendance at primary care with dysmenorrhoea, prevalence of attending primary care with dysmenorrhoea will be stratified by age, region, quintile of multiple deprivation index and ethnicity
**B**	What is the risk of a diagnosis of endometriosis for women recorded as having dysmenorrhoea in adolescence in their primary care record?<br><br>What is the risk of subfertility, chronic pain and ongoing dysmenorrhoea for women recorded as having dysmenorrhoea in adolescence in their primary care records?<br><br>Does prescribed treatment with hormonal therapies affect the risk of a diagnosis of endometriosis following documented adolescent dysmenorrhoea in the primary care record?	prospective open cohort study	Female at birth individuals aged 10-19 in any year during 1.1.2000 to 30.6.2021 who are registered with eligible QResearch practices contributing data to the QResearch database	Five times this number of age-matched randomly selected adolescents who are not recorded as having attended primary care with dysmenorrhoea	dysmenorrhoea documented in the primary care record in adolescence.	Diagnosis of endometriosis at any age during the study period, defined either within the primary care or hospital record.	Diagnosis of adenomyosis, evidence of ongoing menstrual pain, a diagnosis of pelvic pain or a diagnosis of sub-fertility. We will investigate these individually and also in combination as composite outcomes.<br><br>We will document the number of prescriptions (hormonal contraception, GnRH analogues, tranexamic acid, analgesics (NSAIDs, opioids, neuropathic pain medications) and referrals (pelvic USS, to secondary care specialist services).<br><br>We will document the mean (SD) and median (IQR) for the time interval between first presentation with symptoms and a diagnosis of endometriosis, chronic pelvic pain or sub-fertility. We will determine whether this is significantly different if hormonal treatment or treatment to suppress menstruation is prescribed.
**C**	What are primary care relevant risk factors for a diagnosis of endometriosis in adolescence?	nested retrospective case-control study	Patients with a diagnosis of endometriosis in adolescence from the cohort of female at birth individuals aged 10-19 in any year during 1.1.2000 to 30.6.2021 who are registered with eligible QResearch practices contributing data to the QResearch database	Each case will be matched by age with five controls with replacement (i.e. a patient not diagnosed with endometriosis).	Individual symptoms or pattern of symptoms preceding a diagnosis of endometriosis in adolescence.	A diagnosis of endometriosis in adolescence (aged 10-19)	We will also explore the number of treatments prescribed in primary care, categorised as follows: hormonal treatments and contraceptives, prescribed non-steroidal anti-inflammatory medication, analgesics, anti-depressants, medication to manage menstrual flow. The number of referrals from primary care to gynaecology, fertility services, pain services, pelvic ultrasound scan.<br><br>We will calculate the time from presentation with symptoms to definitive diagnosis with endometriosis and determine whether this is different between those prescribed hormonal medication or contraception (documented categorically by number of prescriptions) during adolescence and those not prescribed hormonal medication during adolescence

For peer review only

Data source and variables:

QResearch is a large, validated database of over 35 million anonymised electronic primary care health records dating back to 1989. It is representative of the English general population. Patient data is compiled at an individual level from GP practices through the Egton Medical Information Systems (EMIS) electronic health record system with linkages to the NHS Digital Hospital Episode Statistics (HES) platform[32].

The variables included in this study represent outcomes, exposures, lifestyle factors and potential co-variates. These are summarised in Table 2. Data extracted from QResearch will be derived using SNOMED-CT code groups. Hospital Episode Statistics data will be derived from the International Classification of Diseases 10th Revision code system (ICD-10) and the Office of Population Censuses and Surveys Classification of Interventions and Procedures version 4 (OPCS4) codes.

The variables selected for inclusion in this study have been derived from published literature about symptoms that might suggest endometriosis including studies which have utilised primary care databases to determine symptom patterns[15-17,26,], and evidence about symptoms in adolescence[5,27,28]. These include gynaecological symptoms, as well as symptoms from the gastro-intestinal tract[18], the urinary tract, and the musculoskeletal system[10, 13,15,17,24,27,28].

We have not included variables which may be relevant risk factors for endometriosis, but where coding in primary care is likely to be incomplete or unreliable. Following expert guidance, we have not included age at menarche, family history of menstrual disorders, or anovulatory cycles (where primary care would not usually be able to ascertain or validate the code). However, we have included irregular cycles which may partially reflect anovulation, but which is a descriptive code and so can be meaningfully recorded within primary care records.

The code groups were developed within QWeb using Snomed-CT, with input for our code group definitions from the published literature and expert consultation.

The Snomed, ICD-10, drug group and OCPS codes contributing data to each of the variable code groups are available at https://www.qresearch.org/data/qcode-group-library/.

Demographic and lifestyle variables will be extracted from the primary care records. Data related to the exposure, and primary and secondary outcomes will be extracted from the GP records, with hospital linked data additionally used for the primary and secondary diagnostic outcomes and referrals as detailed below.

Table 2: Variables included in this study

**Type of variable:**		**Project relevant to:**	**Included variables:**	**Variable source**
Demographic variables		A, B, C	Geographical region in England (categorical, 10 regions)<br>Townsend deprivation quintile<br>Ethnicity (categorical; White, Indian, Bangladeshi, Pakistani, Chinese, other Asian, Caribbean, Black African, other)	QResearch
		A, B, C	Age (continuous variable)	QResearch
Lifestyle variables		A, B, C	Smoking status (categorical; non-smoker, ex-smoker, light smoker<10/day, moderate smoker 10-19/day, Heavy smoker >20/day)<br>Body mass index (continuous)	QResearch
Primary exposure		B	Dysmenorrhoea (primary, secondary, not specified, menstrual pain))	QResearch
		C	The pattern of symptoms recorded in the primary care record prior to a diagnosis of adolescent endometriosis.	QResearch
Primary outcome		A	number of adolescents coded with dysmenorrhoea in primary care	QResearch
		B	Diagnosis of endometriosis at any time within the study period	QResearch and HES

For peer review only

		C	Cases are those diagnosed with endometriosis during adolescence	QResearch
Secondary outcomes		A	Demographic variables above	QResearch
		B	Sub-fertility and infertility<br>Pelvic pain<br>Adenomyosis.<br>On-going menstrual pain (all dysmenorrhoea codes as above).	QResearch and HES (sub-fertility, adenomyosis)
		B	Medication and referrals as detailed below	QResearch
Secondary (other) exposures of interest: Medications prescribed in primary care		B, C	Hormonal treatments and contraceptives:<br>• Combined hormonal contraception<br>• Progesterone only contraceptive pills<br>• Intra-Uterine System (IUS) prescriptions issued by GP practice or coded within primary care records<br>• Sub-dermal contraception codes<br>• Gn-rh analogues (prescriptions issued by GP practice)<br>Prescribed non-steroidal anti-inflammatory medication (NSAID)<br>• naproxen, ibuprofen, xox-2 inhibitors<br>• mefanemic acid,<br>Analgesics<br>• codeine, opioids<br>• pregabalin,<br>• gabapentin,<br>Anti-depressants<br>• (SSRI).<br>• Amitryptilline, nortryptilline (due to indication for neuropathic analgesia)<br>Medications to manage menstrual flow:<br>• tranexamic acid	QResearch
Referrals made		B, C	Referrals from primary care to:<br>• Gynaecology<br>• Fertility services<br>• Pain services<br>• Pelvic ultrasound scan<br>Within secondary care:<br>• Pelvic MRI and pelvic ultrasound scan<br>• Fertility treatment<br>• Diagnostic laparoscopy	QResearch<br>OPCS<br>ICD-10
	**Potential confounders or co-variates**			
Gynaecologic variables		B, C	Dyspareunia (superficial, deep, not otherwise specified)<br>Post-coital bleeding<br>Polycystic Ovarian Syndrome (PCOS)<br>Menorrhagia and heavy menstrual bleeding.<br>Dysfunctional uterine bleeding<br>Irregular menstrual cycles (irregular, infrequent or frequent bleeding)<br>Pelvic inflammatory disease<br>Ovarian cysts<br>Congenital uterine or genital tract anomalies (developmental disorders of the female genital tract)<br>Genital pain<br>Vaginismus<br>Amenorrhoea<br>Premenstrual symptoms<br>Vaginal discharge<br>Ovulation pain<br>Menstrual disorder, not otherwise specified	QResearch and HES (for ovarian cysts, PID, congenital uterine or genital tract anomalies))
Non-gynaecologic variables		B, C	Depression<br>Anxiety<br>Irritable bowel syndrome<br>Diarrhoea<br>Constipation<br>Nausea and vomiting<br>dysuria<br>Cystitis and UTI (chronic, recurrent, simple)<br>Rectal or anal pain	QResearch

For peer review only

			Dyschezia (difficult or painful defecation)<br>Rectal bleeding<br>Migraine<br>Low back pain<br>Fatigue<br>Bloating<br>Fibromyalgia	
Pregnancy related outcomes		B	Pregnancy<br>Live birth<br>Ectopic pregnancy	QResearch and HES

Study period:

For the cohort and case control studies (B, C) the study period is from 1.1.2000 to 30.6.2021, the most recent date for which complete data is expected. For study A, the study period is from 1.1.2011 to 30.6.2021.

Inclusion criterion:

We will use the World Health Organisation definition of adolescence, which is 10-19 years old[33].

For all components of the study the inclusion criteria are female at birth individuals aged 10-19 in any year during the study period and who are registered with practices contributing data to the QResearch database. Additional inclusion rules for the different study components are described within individual project method sections.

Exclusion criterion:

Individuals who are not female at birth or not aged 10-19 years during the study period.

Method and statistics plan: Project A

Research Question: What is the prevalence of recorded dysmenorrhoea in adolescents accessing primary care?

Study Design: Descriptive study

Inclusion: individuals aged 10-19 in any year between 1.1.2011-30.6.2021.

Exclusion criterion: No additional rules.

Outcomes: A primary care diagnosis of dysmenorrhoea.

Exposure: Not applicable

Confounding variables: Outcomes will be stratified by the following demographic variables: age, region, quintile of multiple deprivation index and ethnicity

Statistical methods: The prevalence of adolescent dysmenorrhoea in primary care will be reported by year of diagnosis and stratified by age, ethnicity, quintile of deprivation, and region.

Method and statistics plan: Project B

Research questions:

- What is the risk of a diagnosis of endometriosis for women with dysmenorrhoea in adolescence recorded in their primary care record?
- What is the risk of subfertility, chronic pain and ongoing dysmenorrhoea for women recorded as having dysmenorrhoea in adolescence in their primary care records?
- Does prescribed treatment with hormonal therapies affect the risk of a diagnosis of endometriosis following documented adolescent dysmenorrhoea in the primary care record?

Study Design: prospective open cohort study.

For peer review only

Inclusion criterion: All adolescents who are recorded as attending primary care with dysmenorrhoea and a comparator cohort comprising five times this number of age-matched, randomly selected adolescents without replacement who are not recorded as having attended primary care with dysmenorrhoea.

Exclusion criterion: no additional rules

Primary outcomes: Diagnosis of endometriosis at any age during the study period, defined either within the primary care or hospital record.

Secondary outcomes: Diagnosis of adenomyosis, evidence of ongoing menstrual pain, a diagnosis of pelvic pain or a diagnosis of sub-fertility. We will investigate these individually and also in combination as composite outcomes.

Exposure: dysmenorrhoea documented in the primary care record in adolescence.

Potential co-variates or confounding variables to be considered:

- Gynaecological variables: dyspareunia (superficial, deep, not otherwise specified), post-coital bleeding, polycystic ovarian syndrome (PCOS), menorrhagia and heavy menstrual bleeding, dysfunctional uterine bleeding, irregular menstrual cycles (infrequent and frequent), pelvic inflammatory disease, ovarian cysts, congenital uterine or genital tract anomalies (developmental disorders of the female genital tract)
- Non-Gynaecological variables: depression, anxiety, irritable bowel syndrome, diarrhoea, constipation, nausea, irritable bladder, dysuria, chronic cystitis, cystitis and urinary tract infection (UTI), rectal or anal pain, dyschezia, rectal bleeding, migraine, low back pain, fatigue, bloating, fibromyalgia
- Pregnancy related variables: pregnancy, live birth, ectopic pregnancy
- Demographic and lifestyle variables: geographical region in England (categorical, 10 regions), Townsend deprivation quintile, ethnicity, age, body mass index (BMI), smoking
- Medication related: hormonal treatments and contraceptives, prescribed NSAID medication, analgesics, anti-depressants, medication to manage menstrual flow (Table 2).Referrals from primary care to gynaecology, fertility services, pain services, pelvic ultrasound scan

Statistical methods: Hazard ratios (HR) and 95% confidence intervals (CI) for each outcome will be estimated using Cox regression with adjustment for pre-specified co-variates in multi-variable models. The proportional hazards assumption will be checked visually using log minus log survival plots for each regression model. Effect estimates for each symptom will be calculated and visualised in forest plots. Because subjects may enter the cohort at different times or may not yet have experienced one or more of the endpoints, we will use a log rank test to compare time to event curves. We will run separate models for each primary and composite outcome.

The time interval between documentation of symptoms in the primary care record and referral to secondary care or receiving a diagnosis of endometriosis will be mapped using Kaplan-Meier plots.

Potential confounders will be accounted for in the Cox model. We will include consideration of other menstrual diagnoses and symptoms, contraceptive use and pelvic inflammatory disease as potential confounders. In light of the exploratory nature of this project, we have included a wide range of potentially relevant co-variates, including gynaecological and non-gynaecological variables. We will document descriptive statistics (frequency and mean/ standard deviation [SD] or median/ inter-quartile range [IQR]) for the number of occurrences of the following variables as detailed in Table 2: hormonal treatments and contraceptives, prescribed non-steroidal anti-inflammatory medication, analgesics, anti-depressants, medication to manage menstrual flow, referrals from primary care to gynaecology, fertility services, pain services, pelvic ultrasound scan.

We will document the mean (SD) and median (IQR) for the time interval between first presentation with symptoms and a diagnosis of endometriosis, chronic pelvic pain or sub-fertility. We will determine whether this is significantly different if hormonal treatment or treatment to suppress menstruation is prescribed.

Method and statistics plan: Project C

Research Question: What are primary care relevant risk factors for a diagnosis of endometriosis in adolescence?

Research design: retrospective nested matched case-control study

For peer review only

Inclusion Criterion: Patients with a diagnosis of endometriosis in adolescence. Each case will be matched by age with five controls with replacement (i.e. a patient not diagnosed with endometriosis).

Exclusion criterion: no additional rules

Outcome/ case definition: Diagnosis of endometriosis in adolescence.

Exposure: Individual symptoms or pattern of symptoms preceding a diagnosis of endometriosis in adolescence.

Potential confounders or co-variates:

- Gynaecological variables: dyspareunia (superficial, deep, not otherwise specified), post-coital bleeding, polycystic ovarian syndrome (PCOS), menorrhagia and heavy menstrual bleeding, dysfunctional uterine bleeding, irregular menstrual cycles, pelvic inflammatory disease, ovarian cysts, congenital uterine or genital tract anomalies (developmental disorders of the female genital tract)
- Non-Gynaecological variables: depression, anxiety, irritable bowel syndrome, diarrhoea, constipation, nausea, irritable bladder, dysuria, bladder pain syndrome/interstitial cystitis, cystitis and urinary tract infection (UTI), rectal or anal pain, dyschezia, rectal bleeding, migraine, low back pain, fatigue, bloating, fibromyalgia
- Demographic and lifestyle variables: geographical region in England (categorical, 10 regions), Townsend deprivation quintile, ethnicity, age, BMI, smoking
- Medication related: hormonal treatments and contraceptives, prescribed NSAID medication, analgesics, anti-depressants, medication to manage menstrual flow (Table 2).
- Referrals from primary care to gynaecology, fertility services, pain services, pelvic ultrasound scan

Statistics plan: Using conditional logistic regression, we will calculate unadjusted and adjusted odds ratios for adolescent endometriosis for identified exposures. We will consider whether there are exposures that alter the likelihood of a diagnosis of adolescent endometriosis. Symptoms with an OR >1.2 for risk of endometriosis with associated 95% CI which does not contain the value 1 will be considered clinically relevant.

We will explore the number of treatments prescribed in primary care, categorised as follows: hormonal treatments and contraceptives, prescribed non-steroidal anti-inflammatory medication, analgesics, anti-depressants, medication to manage menstrual flow. The number of referrals from primary care to gynaecology, fertility services, pain services, pelvic ultrasound scan.

We will calculate the time from presentation with symptoms to definitive diagnosis with endometriosis and determine whether this is different between those prescribed hormonal medication or contraception (documented categorically by number of prescriptions) during adolescence and those not prescribed hormonal medication during adolescence

Sample size calculation:

We have not been able to identify any published evidence about the incidence or prevalence of coded dysmenorrhoea in adolescence in primary care in the UK or worldwide. Therefore, project A will generate information which will help us refine our calculations of feasibility and necessary parameters for the subsequent studies. Recognising the lack of established recording prevalence, we will include every case within the QResearch database to optimise the likelihood of attaining statistical power.

In recognition of this uncertainty, we have adopted a conservative approach to our sample size calculation and have conducted a formal statistical power and feasibility calculation.

For project B, estimating a conservative hazard ratio of 1.2 for developing one of the primary endpoints, with significance of 0.05 and power of 0.8, we estimate that we would need we would need at least 945 patients with each diagnosis recorded and a comparator cohort of at least 15,742 controls. We consider this to be feasible, given the size of the database.

For project C, we calculate that we would need 2,873 cases with an exposure probability in the control group of 0.1 (or 5,376 cases for an exposure probability of 0.05) to estimate an OR of 1.2 with significance of 0.05 and power of

For peer review only

0.8, considering a matching of 1:5. We consider this to be feasible based on the number of adolescents documented with endometriosis in previous UK based population cohort studies.

Plan for missing data.

We anticipate missing data for ethnicity, body mass index, and smoking status. We will assume that these data are missing at random.

Missing values will be imputed using multiple imputation with chained equations using all outcome and exposure variables in the model. Five imputations will be carried out and Rubin's rules will be used to pool model estimates across the imputed datasets.

Sensitivity analysis

1. Complete vs imputed dataset

We will do a complete-case analysis as sensitivity analysis to check the missing at random (MAR) assumption. Results from both multiple imputation and complete case analyses will be reported.

2. Estimation of the rate ratio of endometriosis diagnosis in adolescence in those with and without documented coding of dysmenorrhoea

Case-control study C explores cases of endometriosis, with controls with and without dysmenorrhoea. We will also conduct a sensitivity analysis within the cohort coded with dysmenorrhoea with cases being those with dysmenorrhoea and adolescent endometriosis, and controls with dysmenorrhoea without diagnosed adolescent endometriosis, to identify other relevant patterns or variables that may affect or predict risk.

Ethics:

The project has been independently peer-reviewed and received ethics approval from the QResearch Scientific Board (reference OX46 under REC 18/EM/0400)[35].

Dissemination:

Our findings will be reviewed with our PPI advisers, who will advise on dissemination and communication approaches. The findings of these studies will be published in peer reviewed academic journals and presented at conferences, including seeking cross-disciplinary avenues for research dissemination. We will work with collaborating advocacy organisations and patient groups to seek opportunities to share what we have learned and consider opportunities for how this can be utilised to improve care.

We will utilise the combined findings to generate an evidence-based infographic to support shared decision making about dysmenorrhoea in community care settings.

Finally, these projects will inform a subsequent qualitative study, exploring adolescent's experiences of menstrual pain. With their input, we will write accessible summaries about important aspects of painful periods which will be freely available on a widely used health experience website (Healthtalk.org).

Discussion:

This study explores the interface between a commonly experienced symptom in adolescence and the likelihood of subsequent diagnoses including endometriosis. By doing this, we seek to identify factors that will help clinicians and patients navigate their care journeys which will likely begin in primary care with presentation with as yet unexplained symptoms. These epidemiological studies will inform a subsequent qualitative study exploring young people's experiences of menstrual pain, including those who have (or have not) sought care, and who do (or do not) have a diagnosis of endometriosis.

The paucity of evidence in this field has led us to propose an initial comparison of cases (all adolescents coded with dysmenorrhoea) against controls without coded dysmenorrhea. Dysmenorrhea is common, affecting approximately 70-90% of the adolescent population. It is not known who, how, and when individuals present to primary care and

For peer review only

we do not know how complete recording or coding is. Therefore it is likely that some of our control group will also experience dysmenorrhoea.

For young people deciding whether to access care, educators, families and front-line gate keeper clinicians, an important initial question is ”how likely is it that any young person with period pain has an underlying condition?”, “What can I advise about long-term outcomes?” and “What can we advise about treatments now? By including all adolescents who either did or did not present with dysmenorrhoea when we explore the likelihood of a diagnosis of endometriosis, means that if there are systemic or structural reasons that affect attendance for care, then we may be able to consider these.

However, knowing how the risk of endometriosis can be stratified among adolescents who do present to primary care with dysmenorrhea is also important. Recognising this, we plan to undertake a sensitivity analysis contrasting rates of endometriosis in adolescents with documented dysmenorrhoea within study C, to see whether there are factors that help identify which adolescents with dysmenorrhea may be more likely to have endometriosis.

Understanding more about which adolescents with dysmenorrhoea to refer for further investigations is critical to improving care journeys. Knowing more about the impacts of treatment with hormonal medications in adolescence is also crucial to inform guidance for adolescents, their families, and health professionals who look after them.

The aims of this project align with health policy within the Women’s Health Strategy which reinforces the need for awareness and resources to support menstrual wellbeing and care for those with endometriosis[36]

This approach will bridge and add to existing literature, which does not focus on the primary care interface crucial to accessing care in the UK health system.

A limitation of this project is its reliance on primary care coding, which may be incomplete or inaccurate. Data entry and recording in primary care is conducted to support clinical care and not predominantly to support research. However, existing understanding of endometriosis symptomatology in primary care is derived using comparable methods, although adolescents are not considered or reported separately[15-17]. Therefore, there is methodological justification for utilising this approach to consider adolescents as proposed in this study.

Limitations include the possibility of bias due to missing data, or from data misclassification, for example miscoding of symptoms or diagnoses. Adolescents with dysmenorrhoea may not have this documented in their primary care record, either because they have not attended for care, not reported this (or been heard), or not had this recorded as a Snomed code (for example if it is documented within consultation free text).

With menstrual symptoms, social acceptability bias may exist and may not be uniformly experienced across the population. The stratified analysis in project A may highlight areas for exploration in the aligned qualitative study. Recorder and recall bias are potentially relevant, whereby symptoms are better coded when a referral or treatment is planned, which will not apply to the comparator group.

Smoking and BMI are more likely to be recorded for young people prescribed hormonal treatment as these prescriptions represent a template driven opportunity to record this data. This data is more likely to be complete for young people prescribed hormonal treatment in primary care (and would not capture this data for those whose hormonal treatment was prescribed in other healthcare settings for example community sexual health clinics). Furthermore, the adolescents using sexual health clinics (rather than primary care) may systematically differ from each other (for example if they vary by location such as rural settings). Patterns of contraception use may also change over time, as demonstrated by the ‘pill scare’ of 1995 which caused a reduction in contraceptive pill use[37]. Menstruation and endometriosis do not link with any QOF codes, although long-acting contraception has done[38], which may act as a driver towards contraception being coded rather than pain or symptoms. Adolescence to adulthood can be a time of mobility, for example if adolescents leave home to travel elsewhere for work or study, which might limit the duration of follow up if people de-register from their GP when they move. Furthermore, these changes may not be uniformly distributed across social or demographic factors.

Finally, adolescent gynaecological units are not uniformly distributed across the country, and access to specialist adolescent services, tertiary or super-specialist care may influence referral rates and patterns of identification and referral[39]. If identified through stratification against region, this could offer a valuable finding.

For peer review only - http://bmjopen.bmj.com/site/about/guidelines.xhtml

We recognise that these represent a significant limitation of this approach. We will perform sensitivity analyses to compare robustness of results between complete-case and imputed analyses.

However, this project proposes a novel approach to develop understanding of a complex and important challenge when moving from symptoms of menstrual pain in adolescence to diagnosis of endometriosis, which is an under-researched and under-recognised health condition, which can have significant adverse impacts on women's health and wellbeing.

For peer review only

COI

KV has received research funding from Bayer Healthcare and honoraria for talks and consultancy fees from Bayer Healthcare, AbbVie and Eli Lilly. The remaining authors declare no relevant COI.

Funding:

This study was funded as part of Sharon Dixon's NIHR Doctoral research fellowship NIHR301787. There is no other relevant funding to declare. The views expressed are those of the author(s) and not necessarily those of the NIHR or the Department of Health and Social Care.

Author Contributions:

SD, KV and J H-C conceived and developed this proposed study. SD, J H-C, TR and MP developed the study design and statistical plan, including sample size calculation and protocol development. SD, J-H-C, KV and JB developed the data extraction plan and study code groups. SD, J H-C, MP, TR, and AS contributed to the proposed plan for data acquisition and curation. SD initially drafted this manuscript. All authors contributed to developing, refining and writing this manuscript.

Acknowledgments:

With thanks to Sue Ziebland, Lisa Hinton and Abigail McNiven for advice and guidance during the development of this project. With enormous thanks to the study PPIE advisers, for their wisdom, encouragement and support.

References:

1. Parker, M.A., Sneddon, A.E. and Arbon, P., 2010. The menstrual disorder of teenagers (MDOT) study: determining typical menstrual patterns and menstrual disturbance in a large population-based study of Australian teenagers. BJOG: An International Journal of Obstetrics & Gynaecology, 117(2), pp.185-192
2. Suvitie, P.A., Hallamaa, M.K., Matomäki, J.M., Mäkinen, J.I. and Perheentupa, A.H., 2016. Prevalence of pain symptoms suggestive of endometriosis among Finnish adolescent girls (TEENMAPS study). Journal of pediatric and adolescent gynecology, 29(2), pp.97-103.
3. Vincenzo De Sanctis, M., Soliman, A., Bernasconi, S., Bianchin, L., Bona, G., Bozzola, M., Buzi, F., De, C., Sanctis, M.D., Tonini, G. and Rigon10, F., 2015. Primary dysmenorrhea in adolescents: prevalence, impact and recent knowledge. Pediatric Endocrinology Reviews (PER), 13(2), pp.465-73
4. Deb S, Raine-Fenning N. Dysmenorrhoea. Obstetrics, Gynaecology & Reproductive Medicine. 2008 Nov 1;18(11):294-9.
5. Smorgick N, Marsh CA, As-Sanie S, Smith YR, Quint EH. Prevalence of pain syndromes, mood conditions, and asthma in adolescents and young women with endometriosis. Journal of pediatric and adolescent gynecology. 2013 Jun 1;26(3):171-5
6. Vincent K, Warnaby C, Stagg CJ, Moore J, Kennedy S, Tracey I. Dysmenorrhoea is associated with central changes in otherwise healthy women. Pain. 2011 Sep 1;152(9):1966-75
7. Johnson, J., 1988. Level of knowledge among adolescent girls regarding effective treatment for dysmenorrhea. Journal of Adolescent Health Care, 9(5), pp.398
8. Armour M, Parry K, Al-Dabbas MA, Curry C, Holmes K, MacMillan F, Ferfolja T, Smith CA. Self-care strategies and sources of knowledge on menstruation in 12,526 young women with dysmenorrhea: A systematic review and meta-analysis. PLoS One. 2019 Jul 24;14(7):e0220103.
9. Houston, A.M., Abraham, A., Huang, Z. and D'Angelo, L.J., 2006. Knowledge, attitudes, and consequences of menstrual health in urban adolescent females. Journal of Pediatric and Adolescent Gynecology, 19(4), pp.271-275

For peer review only

10. De Sanctis V, Matalliotakis M, Soliman AT, Elsefdy H, Di Maio S, Fiscina B. A focus on the distinctions and current evidence of endometriosis in adolescents. Best Practice & Research Clinical Obstetrics & Gynaecology. 2018 Aug 1;51:138-50.
11. Pugsley, Z. and Ballard, K., 2007. Management of endometriosis in general practice: the pathway to diagnosis. Br J Gen Pract, 57(539), pp.470-476.
12. Johnston, J.L., Reid, H. and Hunter, D., 2015. Diagnosing endometriosis in primary care: clinical update. Br J Gen Pract, 65(631), pp.101-102#
13. Hirsch M, Dhillon-Smith R, Cutner A, Yap M, Creighton SM. The prevalence of endometriosis in adolescents with pelvic pain: A systematic review. Journal of Pediatric and Adolescent Gynecology. 2020 Jul 29. 24
14. Brosens, I., Gordts, S. and Benagiano, G., 2013. Endometriosis in adolescents is a hidden, progressive and severe disease that deserves attention, not just compassion. Human Reproduction, 28(8), pp.2026-2031
15. Ballard, K.D., Seaman, H.E., De Vries, C.S. and Wright, J.T., 2008. Can symptomatology help in the diagnosis of endometriosis? Findings from a national case–control study—Part 1. BJOG: An International Journal of Obstetrics & Gynaecology, 115(11), pp.1382-1391.
16. Cea Soriano, L., López-Garcia, E., Schulze-Rath, R. and Garcia Rodríguez, L.A., 2017. Incidence, treatment and recurrence of endometriosis in a UK-based population analysis using data from The Health Improvement Network and the Hospital Episode Statistics database. The European Journal of Contraception & Reproductive Health Care, 22(5), pp.334-343.
17. Burton, C., Iversen, L., Bhattacharya, S., Ayansina, D., Saraswat, L. and Sleeman, D., 2017. Pointers to earlier diagnosis of endometriosis: a nested case-control study using primary care electronic health records. Br J Gen Pract, 67(665), pp.e816-e823.
18. Ghai V, Jan H, Shakir F, Haines P, Kent A. Diagnostic delay for superficial and deep endometriosis in the United Kingdom. Journal of Obstetrics and Gynaecology. 2020 Jan 2;40(1):83-9.
19. Sieberg CB, Lunde CE, Borsook D. Endometriosis and pain in the adolescent-striking early to limit suffering: A narrative review. Neuroscience & Biobehavioral Reviews. 2020 Jan 1;108:866-76.
20. Janssen, E.B., Rijkers, A.C.M., Hoppenbrouwers, K., Meuleman, C. and d'Hooghe, T.M., 2013. Prevalence of endometriosis diagnosed by laparoscopy in adolescents with dysmenorrhea or chronic pelvic pain: a systematic review. Human reproduction update, 19(5), pp.570-582
21. Shim JY, Laufer MR. Adolescent endometriosis: an update. Journal of pediatric and adolescent gynecology. 2020 Apr 1;33(2):112-9.
22. Guideline of European Society of Human Reproduction and Embryology Endometriosis, https://www.eshre.eu/Guideline/Endometriosis, accessed 30.9.22
23. Chapron, C., Querleu, D., Bruhat, M.A., Madelenat, P., Fernandez, H., Pierre, F. and Dubuisson, J.B., 1998. Surgical complications of diagnostic and operative gynaecological laparoscopy: a series of 29,966 cases. Human reproduction (Oxford, England), 13(4), pp.867-872. 46.
24. Shah, D.K. and Missmer, S.A., 2011. Scientific investigation of endometriosis among adolescents. Journal of pediatric and adolescent gynecology, 24(5), pp.S18-S19
25. Clemenza S, Vannuccini S, Capezzuoli T, Meleca CI, Pampaloni F, Petraglia F. Is primary dysmenorrhea a precursor of future endometriosis development?. Gynecological Endocrinology. 2021 Apr 3;37(4):287-93.
26. Sarıdoğan, E., 2017. Adolescent endometriosis. European Journal of Obstetrics & Gynecology and Reproductive Biology, 209, pp.46-49
27. DiVasta, A.D., Vitonis, A.F., Laufer, M.R. and Missmer, S.A., 2018. Spectrum of symptoms in women diagnosed with endometriosis during adolescence vs adulthood. American journal of obstetrics and gynecology, 218(3), pp.324)
28. Yeung Jr P, Gupta S, Gieg S. Endometriosis in adolescents: a systematic review. Journal of Endometriosis and Pelvic Pain Disorders. 2017 Jan;9(1):17-29.
29. Randhawa AE, Tufte-Hewett AD, Weckesser AM, Jones GL, Hewett FG. Secondary School Girls' Experiences of Menstruation and Awareness of Endometriosis: A Cross-Sectional Study. Journal of pediatric and adolescent gynecology. 2021 Oct 1;34(5):643-8.
30. Williams CE, Creighton SM. Menstrual disorders in adolescents: review of current practice. Hormone research in Paediatrics. 2012;78(3):135-43.
31. Knox B, Ong YC, Bakar MA, Grover SR. A longitudinal study of adolescent dysmenorrhoea into adulthood. European journal of pediatrics. 2019 Sep 1;178(9):1325-32

For peer review only

32. QResearch, Home - QResearch, accessed 30.9.22
33. World Health Organisation, Adolescent health, https://www.who.int/health-topics/adolescent-health
34. Dixon S, McNiven A, Talbot A, Hinton L. Navigating possible endometriosis in primary care: a qualitative study of GP perspectives. British Journal of General Practice. 2021 Sep 1;71(710):e668-76.
35. What knowledge would support improving the management of teenagers with dysmenorrhoea in primary care? - QResearch, accessed 30.9.22
36. Women's health Strategy for England Policy paper, Women's Health Strategy for England - GOV.UK (www.gov.uk), accessed 30.9.22
37. Furedi A. The public health implications of the 1995 'pill scare'. Hum Reprod Update. 1999 Nov-Dec;5(6):621-6. doi: 10.1093/humupd/5.6.621. PMID: 10652971
38. Pasvol TJ, Macgregor EA, Rait G, Horsfall L. Time trends in contraceptive prescribing in UK primary care 2000–2018: a repeated cross-sectional study. BMJ sexual & reproductive health. 2022 Jul 1;48(3):193-8.
39. The British Society for Paediatric and Adolescent Gynaecology UK map of services, UK Map of Services – BritSPAG, accessed 30.9.22

For peer review only

Figure 1: Schematic representation of the research aim and questions.

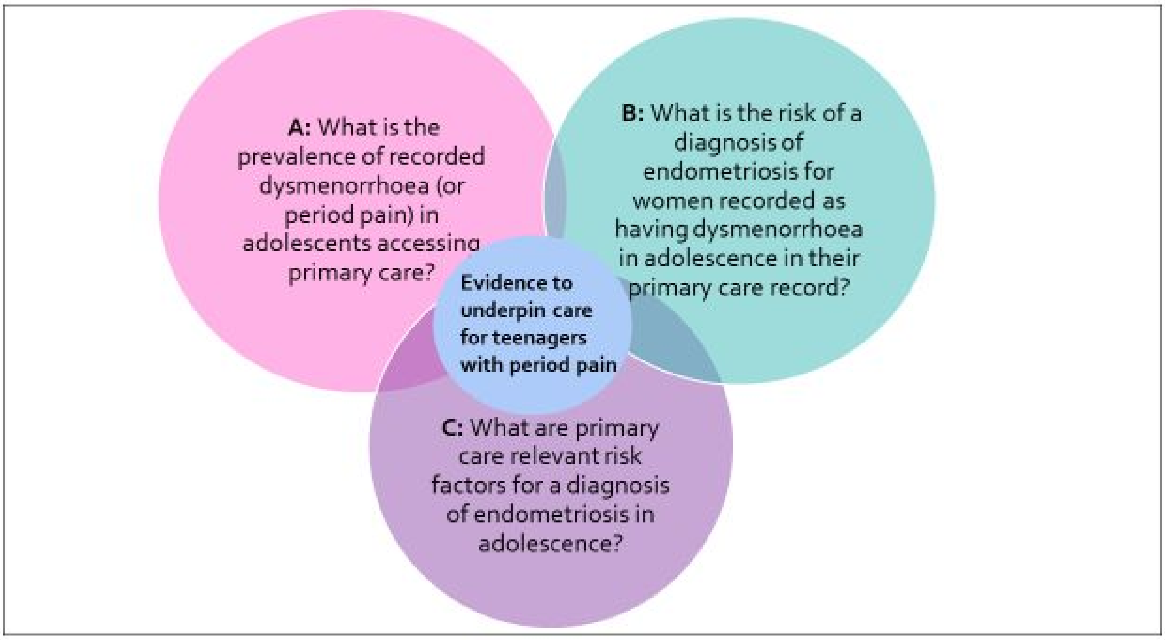

For peer review only

BMJ Open

Exploring the interface between adolescent dysmenorrhoea and endometriosis: A protocol for a cohort and nested case-control study within the QResearch Database

Journal:	*BMJ Open*
Manuscript ID	bmjopen-2022-069984.R1
Article Type:	Protocol
Date Submitted by the Author:	21-Dec-2022
Complete List of Authors:	Dixon, Sharon; University of Oxford, Nuffield Department of Primary Care Health Sciences<br>Ranger, Tom; Oxford University, Primary Care Health Sciences<br>Burchardt, Judith; Oxford University, Primary Care Health Sciences<br>Patone , Martina ; University of Oxford,<br>Snelling, Andrew; Oxford University, Nuffield Department of Primary Care Health Sciences<br>Vincent, Katy; Oxford University, Nuffield Department of Women's and Reproductive Health<br>Hippisley-Cox, Julia; University of Oxford, Nuffield Department of Primary Care Sciences
**Primary Subject Heading**:	Epidemiology
Secondary Subject Heading:	General practice / Family practice, Reproductive medicine, Sexual health
Keywords:	EPIDEMIOLOGY, GYNAECOLOGY, PRIMARY CARE

SCHOLARONE™
Manuscripts

Exploring the interface between adolescent dysmenorrhoea and endometriosis: A protocol for a cohort and nested case-control study within the QResearch Database

*Sharon Dixon[1], Tom Ranger[1], Judith Burchardt[1], Martina Patone[1], Andrew Snelling[1], Katy Vincent[2], Julia Hippisley-Cox[1]

1= Nuffield Department of Primary Care Health Sciences, University of Oxford

2=Nuffield Department of Women's and Reproductive Healthcare, University of Oxford.

Corresponding author:

Sharon Dixon

Radcliffe Primary Care Building,

Radcliffe Observatory Quarter,

Woodstock Road, Oxford. OX2 6GG.

Tel: +44 1865 617855

Funding source:

This study was funded as part of Sharon Dixon's NIHR Doctoral research fellowship NIHR301787. There is no other relevant funding to declare.

Disclaimer:

The views expressed are those of the author(s) and not necessarily those of the NIHR or the Department of Health and Social Care.

COI:

KV has received research funding from Bayer Healthcare and honoraria for talks and consultancy fees from Bayer Healthcare, AbbVie and Eli Lilly. The remaining authors declare no relevant COI

Word count: 4043

Number of tables: 2

Number of figures: 1

Number of references: 39

Sharon Dixon sharon.dixon@phc.ox.ac.uk

For peer review only

Abstract:

Introduction:

Dysmenorrhoea affects approximately 70-91% of adolescents who menstruate, with approximately one third experiencing severe symptoms with impacts on education, work and leisure. Dysmenorrhoea can occur without identifiable pathology, but can indicate underlying conditions, including congenital genital tract anomalies or endometriosis. There is a need for evidence about the management and incidence of dysmenorrhoea in primary care, the impact of treatments in adolescence on long-term outcomes, and when to consider the possibility of endometriosis in adolescence.

Methods and analysis:

This study aims to improve the evidence base for adolescents presenting to primary care with dysmenorrhoea. It comprises three inter-linked studies. Utilising the QResearch database, the study population includes all female at birth participants aged 10-19 any time between 1.1.2000 and 30.6.2021. We will undertake (i) a descriptive study documenting the prevalence of coded dysmenorrhoea in primary care, stratified by demographic variables, reported using descriptive statistics; (ii) a prospective open cohort study following an index cohort of all adolescents recorded as attending primary care with dysmenorrhoea and a comparator cohort of five times as many who have not, to determine the hazard ratio for a diagnosis of endometriosis, adenomyosis, ongoing menstrual pain or sub-fertility (considered singly and in combination) anytime during the study period; and (iii) a nested case-control study for adolescents diagnosed with endometriosis, using conditional logistic regression, to determine the odds ratio for symptom(s) preceding this diagnosis.

Ethics and Dissemination:

The project has been independently peer-reviewed and received ethics approval from the QResearch Scientific Board (reference OX46 under REC 18/EM/0400).

In addition to publication in peer reviewed academic journals, we will utilise the combined findings to generate a resource and infographic to support shared decision-making about dysmenorrhoea in community health settings. Additionally, the findings will be used to inform a subsequent qualitative study, exploring adolescent's experiences of menstrual pain.

Strengths and limitations:

- Focussing on the adolescent period will add nuance to existing epidemiological studies utilising primary care databases exploring the interface between presentation with dysmenorrhoea and diagnosis with endometriosis.
- We will be able to interrogate the findings of this study with a subsequent qualitative study, which will allow exploration and context for questions identified in this research.
- This project is dependent on primary care coding, which may be incomplete. We can explore coded dysmenorrhoea, but this may not represent all presentations with dysmenorrhoea, and some of the variables in our analysis are not linked to structural drivers for recording, such as the QOF framework.
- Recognition of adolescent endometriosis has likely evolved throughout the study period. We will be able to document and describe this, but it could influence inferences about risk.

For peer review only

Introduction:

Dysmenorrhoea is common amongst teenagers who menstruate, with prevalence rates of approximately 70- 91%[1]. Moreover, approximately a third of these teenagers experience severe dysmenorrhoea[2], which is a significant cause of school[3] and workplace absence,[4] and is associated with mood disorders and other chronic pain syndromes[5]. Furthermore, the experience of dysmenorrhoea itself is associated with changes to the central nervous system, including an increased sensitivity to noxious stimuli[6]. Despite the existence of therapeutic interventions that can help treat pain, community surveys suggest that many young people with period pain do not seek medical help or advice[7-9], which highlights the importance of developing resources and services supporting the recognition and treatment of pain.

Dysmenorrhoea can occur in the absence of identifiable underlying pathology (termed primary dysmenorrhoea) or be associated with other conditions, including endometriosis[10]. Endometriosis occurs in approximately 2-10% of adult women and is associated with sub-fertility and chronic pelvic pain[11-13]. Adolescent endometriosis is believed to be under-recognised[10,14], however many women diagnosed with endometriosis in adulthood report symptom onset in adolescence[14]. Furthermore, there are documented significant delays for adolescents and adults between presentation with symptoms and receiving a diagnosis of endometriosis[15-18] and there are concerns that delay in diagnosis contributes to disease progression[13,14,19]. Therefore it is critical to understand possible earlier clinical signs and symptoms.

The currently published evidence about the incidence of adolescent endometriosis comes predominantly from tertiary or specialised surgical gynaecological units. [20,21]. There is less knowledge of the prevalence or incidence in the community[21]. For front line primary care clinicians working with this tertiary care estimate risks a denominator problem because the incidence in the community is likely to be significantly lower than in people presenting to specialist clinics. Without knowing what percentage of young people are seen and then referred for onward specialist care for severe dysmenorrhoea, this risk or incidence can be difficult to utilise, contextualise, or communicate in community settings. This is important because the only way to definitively rule out endometriosis is with a laparoscopy[22], a procedure associated with risk[23].

Uncertainties or unknowns in adolescent endometriosis include the lack of long-term outcome data, and whether interventions in teenagers influence long-term outcomes[21,24]. Alongside the uncertainty about prevalence, there is also uncertainty about whether symptom and menstrual cycle suppression with hormonal treatment might offer potential reductions in the risk of both long- and short-term outcomes of endometriosis [24,25] or whether this instead risks masking symptoms, contributes to endometriosis progression, or delays diagnosis, potentially leading to an increased risk of adverse outcomes[18,25,26] .

There is evidence that adolescents with endometriosis may present with different symptom patterns from adult women[27,28].

We are aware of no evidence about the incidence, management, or long-term outcomes of teenagers seen in UK primary care with dysmenorrhoea. A recent survey of 442 secondary school students in England reported that 93.6% experienced period pain and 30% of those who replied to the survey had seen their GP[29].

In the existing research which has used routinely collected health data to understand patterns of presentation and journeys to diagnosis for endometriosis from UK primary care, findings about adolescents have not been reported separately[15-17]. In this study, we will use comparable methods to those utilised to delineate risk and symptomatic features suggesting endometriosis in adult women, but focus specifically on adolescents.

The co-variates included in this study are derived from our review of the existing epidemiological literature[27], including extrapolation from studies on adult women[15-17], and our expert clinical experience, including clinicians working in primary care and adolescent gynaecology. There is very little adolescent specific (or primary care) evidence or resources to support adolescents with menstrual pain seeking advice and treatment[30]. There is uncertainty about who to refer, or when, and there is a need for evidence about the likelihood of associated conditions or on-going pain i.e. the natural history of adolescent dysmenorrhoea[24,31.] These are the questions which this research seeks to address.

For peer review only

Research aims:

The over-arching research aim is to improve the evidence base underpinning the diagnosis and care for teenagers with dysmenorrhoea. This study addresses three related but separate research questions, which interface to inform the central research aim:

A: What is the prevalence of recorded dysmenorrhoea (or period pain) in adolescents accessing primary care?

B: What is the risk of a diagnosis of endometriosis for women with dysmenorrhoea in adolescence recorded in their primary care record?

C: What are primary care relevant risk factors for a diagnosis of endometriosis in adolescence?

The research objectives for these three inter-linked epidemiologic studies are represented schematically in figure 1.

Figure 1: Schematic representation of the research aim and questions.

Methods and analysis:

Patient and Public Involvement and Engagement (PPIE):

This project has been developed with PPI guidance from teenagers and women with lived experience of dysmenorrhoea, including those with and without a diagnosis of endometriosis. Understanding how teenagers with menstrual difficulties can best be supported in primary care emerged as an important research and service need from our previous qualitative research with GPs which included reporting PPI feedback on this work from women with lived experience of endometriosis and menstrual pain[32]. Questions included when to consider endometriosis, when to refer for investigations, and whether symptom suppression with hormonal contraception represented adequate care.

The PPI advisers for this study include adolescents and young adults with lived experience of menstrual pain and adults with experience of pain in adolescence, and input from endometriosis UK. They helped identify the questions which this study seeks to address, including the focus on presentation with symptoms, the interface between symptoms and possible diagnosis of endometriosis, knowing more about the possible long-term outcomes of adolescent dysmenorrhoea, and whether treatment with hormonal contraception helps (or not).

They valued the approach of both moving prospectively from symptoms to diagnosis, alongside looking retrospectively from diagnosis to symptoms. They were interested in exploring the demographic stratification in project A, to consider whether there are any patterns that might suggest unmet needs.

Throughout this study, we will regularly meet with our PPI advisers, and seek their guidance and insights in helping us make sense of our findings, whether they suggest emerging questions, and consider how we can best share and utilise our results to improve knowledge and care.

Study design:

This study comprises three inter-linked population-based projects (designated A, B, C) conducted within the QResearch database[32]: project A is a descriptive study, project B is a prospective open cohort study and project C is a retrospective nested matched case-control study.

The project components are described individually in turn below and are summarised in Table 1.

For peer review only

Table1: Summary table describing the three study epidemiology components:

Project	Project research question(s)	Study design	Population included	Comparators /control	Exposure	Primary outcome(s)	Secondary Outcome(s)
A	What is the prevalence of recorded dysmenorrhoea (or period pain) in adolescents accessing primary care?	Descriptive study	Female at birth individuals aged 10-19 in any year during 1.1.2011 to 30.6.2021 who are registered with eligible QResearch practices contributing data to the QResearch database	None	Not applicable	A diagnosis of dysmenorrhoea in primary care.	To identify whether there are disparities in recorded attendance at primary care with dysmenorrhoea, prevalence of attending primary care with dysmenorrhoea will be stratified by age, region, quintile of multiple deprivation index and ethnicity
B	What is the risk of a diagnosis of endometriosis for women recorded as having dysmenorrhoea in adolescence in their primary care record?<br><br>What is the risk of subfertility, chronic pain and ongoing dysmenorrhoea for women recorded as having dysmenorrhoea in adolescence in their primary care records?<br><br>Does prescribed treatment with hormonal therapies affect the risk of a diagnosis of endometriosis following documented adolescent dysmenorrhoea in the primary care record?	prospective open cohort study	Female at birth individuals aged 10-19 in any year during 1.1.2000 to 30.6.2021 who are registered with eligible QResearch practices contributing data to the QResearch database	Five times this number of age-matched randomly selected adolescents who are not recorded as having attended primary care with dysmenorrhoea	dysmenorrhoea documented in the primary care record in adolescence.	Diagnosis of endometriosis at any age during the study period, defined either within the primary care or hospital record.	Diagnosis of adenomyosis, evidence of ongoing menstrual pain, a diagnosis of pelvic pain or a diagnosis of sub-fertility. We will investigate these individually and also in combination as composite outcomes.<br><br>We will document the number of prescriptions (hormonal contraception, GnRH analogues, tranexamic acid, analgesics (NSAIDs, opioids, neuropathic pain medications) and referrals (pelvic USS, to secondary care specialist services).<br><br>We will document the mean (SD) and median (IQR) for the time interval between first presentation with symptoms and a diagnosis of endometriosis, chronic pelvic pain or sub-fertility. We will determine whether this is significantly different if hormonal treatment or treatment to suppress menstruation is prescribed.
C	What are primary care relevant risk factors for a diagnosis of endometriosis in adolescence?	nested retrospective case-control study	Patients with a diagnosis of endometriosis in adolescence from the cohort of female at birth individuals aged 10-19 in any year during 1.1.2000 to 30.6.2021 who are registered with eligible QResearch practices contributing data to the QResearch database	Each case will be matched by age with five controls with replacement (i.e. a patient not diagnosed with endometriosis).	Individual symptoms or pattern of symptoms preceding a diagnosis of endometriosis in adolescence.	A diagnosis of endometriosis in adolescence (aged 10-19)	We will also explore the number of treatments prescribed in primary care, categorised as follows: hormonal treatments and contraceptives, prescribed non-steroidal anti-inflammatory medication, analgesics, anti-depressants, medication to manage menstrual flow. The number of referrals from primary care to gynaecology, fertility services, pain services, pelvic ultrasound scan.<br><br>We will calculate the time from presentation with symptoms to definitive diagnosis with endometriosis and determine whether this is different between those prescribed hormonal medication or contraception (documented categorically by number of prescriptions) during adolescence and those not prescribed hormonal medication during adolescence

For peer review only

Data source and variables:

QResearch is a large, validated database of over 35 million anonymised electronic primary care health records dating back to 1989. It is representative of the English general population. Patient data is compiled at an individual level from GP practices through the Egton Medical Information Systems (EMIS) electronic health record system with linkages to the NHS Digital Hospital Episode Statistics (HES) platform[33].

The variables included in this study represent outcomes, exposures, lifestyle factors and potential co-variates. These are summarised in Table 2. Data extracted from QResearch will be derived using SNOMED-CT code groups. Hospital Episode Statistics data will be derived from the International Classification of Diseases 10th Revision code system (ICD-10) and the Office of Population Censuses and Surveys Classification of Interventions and Procedures version 4 (OPCS4) codes.

The variables selected for inclusion in this study have been derived from published literature about symptoms that might suggest endometriosis including studies which have utilised primary care databases to determine symptom patterns[15-17,26,], and evidence about symptoms in adolescence[5,27,28]. These include gynaecological symptoms, as well as symptoms from the gastro-intestinal tract[18], the urinary tract, and the musculoskeletal system[10, 13,15,17,24,27,28].

We have not included variables which may be relevant risk factors for endometriosis, but where coding in primary care is likely to be incomplete or unreliable. Following expert guidance, we have not included age at menarche, family history of menstrual disorders, or anovulatory cycles (where primary care would not usually be able to ascertain or validate the code). However, we have included irregular cycles which may partially reflect anovulation, but which is a descriptive code and so can be meaningfully recorded within primary care records.

The code groups were developed within QWeb using Snomed-CT, with input for our code group definitions from the published literature and expert consultation.

The Snomed, ICD-10, drug group and OCPS codes contributing data to each of the variable code groups are available at https://www.qresearch.org/data/qcode-group-library/.

Demographic and lifestyle variables will be extracted from the primary care records. Data related to the exposure, and primary and secondary outcomes will be extracted from the GP records, with hospital linked data additionally used for the primary and secondary diagnostic outcomes and referrals as detailed below.

Table 2: Variables included in this study

**Type of variable:**		**Project relevant to:**	**Included variables:**	**Variable source**
Demographic variables		A, B, C	Geographical region in England (categorical, 10 regions)<br>Townsend deprivation quintile<br>Ethnicity (categorical; White, Indian, Bangladeshi, Pakistani, Chinese, other Asian, Caribbean, Black African, other)	QResearch
		A, B, C	Age (continuous variable)	QResearch
Lifestyle variables		A, B, C	Smoking status (categorical; non-smoker, ex-smoker, light smoker<10/day, moderate smoker 10-19/day, Heavy smoker >20/day)<br>Body mass index (continuous)	QResearch
Primary exposure		B	Dysmenorrhoea (primary, secondary, not specified, menstrual pain))	QResearch
		C	The pattern of symptoms recorded in the primary care record prior to a diagnosis of adolescent endometriosis.	QResearch
Primary outcome		A	number of adolescents coded with dysmenorrhoea in primary care	QResearch
		B	Diagnosis of endometriosis at any time within the study period	QResearch and HES

For peer review only

		C	Cases are those diagnosed with endometriosis during adolescence	QResearch
Secondary outcomes		A	Demographic variables above	QResearch
		B	Sub-fertility and infertility<br>Pelvic pain<br>Adenomyosis.<br>On-going menstrual pain (all dysmenorrhoea codes as above).	QResearch and HES (sub-fertility, adenomyosis)
		B	Medication and referrals as detailed below	QResearch
Secondary (other) exposures of interest: Medications prescribed in primary care		B, C	Hormonal treatments and contraceptives:<br>• Combined hormonal contraception<br>• Progesterone only contraceptive pills<br>• Intra-Uterine System (IUS) prescriptions issued by GP practice or coded within primary care records<br>• Sub-dermal contraception codes<br>• Gn-rh analogues (prescriptions issued by GP practice)<br>Prescribed non-steroidal anti-inflammatory medication (NSAID)<br>• naproxen, ibuprofen, xox-2 inhibitors<br>• mefanemic acid,<br>Analgesics<br>• codeine, opioids<br>• pregabalin,<br>• gabapentin,<br>Anti-depressants<br>• (SSRI).<br>• Amitryptilline, nortryptilline (due to indication for neuropathic analgesia)<br>Medications to manage menstrual flow:<br>• tranexamic acid	QResearch
Referrals made		B, C	Referrals from primary care to:<br>• Gynaecology<br>• Fertility services<br>• Pain services<br>• Pelvic ultrasound scan<br>Within secondary care:<br>• Pelvic MRI and pelvic ultrasound scan<br>• Fertility treatment<br>• Diagnostic laparoscopy	QResearch<br>OPCS<br>ICD-10
	**Potential confounders or co-variates**			
Gynaecologic variables		B, C	Dyspareunia (superficial, deep, not otherwise specified)<br>Post-coital bleeding<br>Polycystic Ovarian Syndrome (PCOS)<br>Menorrhagia and heavy menstrual bleeding.<br>Dysfunctional uterine bleeding<br>Irregular menstrual cycles (irregular, infrequent or frequent bleeding)<br>Pelvic inflammatory disease<br>Ovarian cysts<br>Congenital uterine or genital tract anomalies (developmental disorders of the female genital tract)<br>Genital pain<br>Vaginismus<br>Amenorrhoea<br>Premenstrual symptoms<br>Vaginal discharge<br>Ovulation pain<br>Menstrual disorder, not otherwise specified	QResearch and HES (for ovarian cysts, PID, congenital uterine or genital tract anomalies))
Non-gynaecologic variables		B, C	Depression<br>Anxiety<br>Irritable bowel syndrome<br>Diarrhoea<br>Constipation<br>Nausea and vomiting<br>dysuria<br>Cystitis and UTI (chronic, recurrent, simple)<br>Rectal or anal pain<br>Dyschezia (difficult or painful defecation)<br>Rectal bleeding<br>Migraine<br>Low back pain	QResearch

For peer review only

			Fatigue<br>Bloating<br>Fibromyalgia	
Pregnancy related outcomes		B	Pregnancy<br>Live birth<br>Ectopic pregnancy	QResearch and HES

Study period:

For the cohort and case control studies (B, C) the study period is from 1.1.2000 to 30.6.2021, the most recent date for which complete data is expected. For study A, the study period is from 1.1.2011 to 30.6.2021.

Inclusion criterion:

We will use the World Health Organisation definition of adolescence, which is 10-19 years old[34].

For all components of the study the inclusion criteria are female at birth individuals aged 10-19 in any year during the study period and who are registered with practices contributing data to the QResearch database. Additional inclusion rules for the different study components are described within individual project method sections.

Exclusion criterion:

Individuals who are not female at birth or not aged 10-19 years during the study period.

Method and statistics plan: Project A

Research Question: What is the prevalence of recorded dysmenorrhoea in adolescents accessing primary care?

Study Design: Descriptive study

Inclusion: individuals aged 10-19 in any year between 1.1.2011-30.6.2021.

Exclusion criterion: No additional rules.

Outcomes: A primary care diagnosis of dysmenorrhoea.

Exposure: Not applicable

Confounding variables: Outcomes will be stratified by the following demographic variables: age, region, quintile of multiple deprivation index and ethnicity

Statistical methods: The prevalence of adolescent dysmenorrhoea in primary care will be reported by year of diagnosis and stratified by age, ethnicity, quintile of deprivation, and region.

Method and statistics plan: Project B

Research questions:

- What is the risk of a diagnosis of endometriosis for women with dysmenorrhoea in adolescence recorded in their primary care record?
- What is the risk of subfertility, chronic pain and ongoing dysmenorrhoea for women recorded as having dysmenorrhoea in adolescence in their primary care records?
- Does prescribed treatment with hormonal therapies affect the risk of a diagnosis of endometriosis following documented adolescent dysmenorrhoea in the primary care record?

Study Design: prospective open cohort study.

Inclusion criterion: All adolescents who are recorded as attending primary care with dysmenorrhoea and a comparator cohort comprising five times this number of age-matched, randomly selected adolescents without replacement who are not recorded as having attended primary care with dysmenorrhoea.

Exclusion criterion: no additional rules

For peer review only

Primary outcomes: Diagnosis of endometriosis at any age during the study period, defined either within the primary care or hospital record.

Secondary outcomes: Diagnosis of adenomyosis, evidence of ongoing menstrual pain, a diagnosis of pelvic pain or a diagnosis of sub-fertility. We will investigate these individually and also in combination as composite outcomes.

Exposure: dysmenorrhoea documented in the primary care record in adolescence.

Potential co-variates or confounding variables to be considered:

- Gynaecological variables: dyspareunia (superficial, deep, not otherwise specified), post-coital bleeding, polycystic ovarian syndrome (PCOS), menorrhagia and heavy menstrual bleeding, dysfunctional uterine bleeding, irregular menstrual cycles (infrequent and frequent), pelvic inflammatory disease, ovarian cysts, congenital uterine or genital tract anomalies (developmental disorders of the female genital tract)
- Non-Gynaecological variables: depression, anxiety, irritable bowel syndrome, diarrhoea, constipation, nausea, irritable bladder, dysuria, chronic cystitis, cystitis and urinary tract infection (UTI), rectal or anal pain, dyschezia, rectal bleeding, migraine, low back pain, fatigue, bloating, fibromyalgia
- Pregnancy related variables: pregnancy, live birth, ectopic pregnancy
- Demographic and lifestyle variables: geographical region in England (categorical, 10 regions), Townsend deprivation quintile, ethnicity, age, body mass index (BMI), smoking
- Medication related: hormonal treatments and contraceptives, prescribed NSAID medication, analgesics, anti-depressants, medication to manage menstrual flow (Table 2).Referrals from primary care to gynaecology, fertility services, pain services, pelvic ultrasound scan

Statistical methods: Hazard ratios (HR) and 95% confidence intervals (CI) for each outcome will be estimated using Cox regression with adjustment for pre-specified co-variates in multi-variable models. The proportional hazards assumption will be checked visually using log minus log survival plots for each regression model. Effect estimates for each symptom will be calculated and visualised in forest plots. Because subjects may enter the cohort at different times or may not yet have experienced one or more of the endpoints, we will use a log rank test to compare time to event curves. We will run separate models for each primary and composite outcome.

The time interval between documentation of symptoms in the primary care record and referral to secondary care or receiving a diagnosis of endometriosis will be mapped using Kaplan-Meier plots.

Potential confounders will be accounted for in the Cox model. We will include consideration of other menstrual diagnoses and symptoms, contraceptive use and pelvic inflammatory disease as potential confounders. In light of the exploratory nature of this project, we have included a wide range of potentially relevant co-variates, including gynaecological and non-gynaecological variables. We will document descriptive statistics (frequency and mean/ standard deviation [SD] or median/ inter-quartile range [IQR]) for the number of occurrences of the following variables as detailed in Table 2: hormonal treatments and contraceptives, prescribed non-steroidal anti-inflammatory medication, analgesics, anti-depressants, medication to manage menstrual flow, referrals from primary care to gynaecology, fertility services, pain services, pelvic ultrasound scan.

We will document the mean (SD) and median (IQR) for the time interval between first presentation with symptoms and a diagnosis of endometriosis, chronic pelvic pain or sub-fertility. We will determine whether this is significantly different if hormonal treatment or treatment to suppress menstruation is prescribed.

Method and statistics plan: Project C

Research Question: What are primary care relevant risk factors for a diagnosis of endometriosis in adolescence?

Research design: retrospective nested matched case-control study

Inclusion Criterion: Patients with a diagnosis of endometriosis in adolescence. Each case will be matched by age with five controls with replacement (i.e. a patient not diagnosed with endometriosis).

Exclusion criterion: no additional rules

Outcome/ case definition: Diagnosis of endometriosis in adolescence.

For peer review only - http://bmjopen.bmj.com/site/about/guidelines.xhtml

Exposure: Individual symptoms or pattern of symptoms preceding a diagnosis of endometriosis in adolescence.

Potential confounders or co-variates:

- Gynaecological variables: dyspareunia (superficial, deep, not otherwise specified), post-coital bleeding, polycystic ovarian syndrome (PCOS), menorrhagia and heavy menstrual bleeding, dysfunctional uterine bleeding, irregular menstrual cycles, pelvic inflammatory disease, ovarian cysts, congenital uterine or genital tract anomalies (developmental disorders of the female genital tract)
- Non-Gynaecological variables: depression, anxiety, irritable bowel syndrome, diarrhoea, constipation, nausea, irritable bladder, dysuria, bladder pain syndrome/interstitial cystitis, cystitis and urinary tract infection (UTI), rectal or anal pain, dyschezia, rectal bleeding, migraine, low back pain, fatigue, bloating, fibromyalgia
- Demographic and lifestyle variables: geographical region in England (categorical, 10 regions), Townsend deprivation quintile, ethnicity, age, BMI, smoking
- Medication related: hormonal treatments and contraceptives, prescribed NSAID medication, analgesics, anti-depressants, medication to manage menstrual flow (Table 2).
- Referrals from primary care to gynaecology, fertility services, pain services, pelvic ultrasound scan

Statistics plan: Using conditional logistic regression, we will calculate unadjusted and adjusted odds ratios for adolescent endometriosis for identified exposures. We will consider whether there are exposures that alter the likelihood of a diagnosis of adolescent endometriosis. Symptoms with an OR >1.2 for risk of endometriosis with associated 95% CI which does not contain the value 1 will be considered clinically relevant.

We will explore the number of treatments prescribed in primary care, categorised as follows: hormonal treatments and contraceptives, prescribed non-steroidal anti-inflammatory medication, analgesics, anti-depressants, medication to manage menstrual flow. The number of referrals from primary care to gynaecology, fertility services, pain services, pelvic ultrasound scan.

We will calculate the time from presentation with symptoms to definitive diagnosis with endometriosis and determine whether this is different between those prescribed hormonal medication or contraception (documented categorically by number of prescriptions) during adolescence and those not prescribed hormonal medication during adolescence

Sample size calculation:

We have not been able to identify any published evidence about the incidence or prevalence of coded dysmenorrhoea in adolescence in primary care in the UK or worldwide. Therefore, project A will generate information which will help us refine our calculations of feasibility and necessary parameters for the subsequent studies. Recognising the lack of established recording prevalence, we will include every case within the QResearch database to optimise the likelihood of attaining statistical power.

In recognition of this uncertainty, we have adopted a conservative approach to our sample size calculation and have conducted a formal statistical power and feasibility calculation.

For project B, estimating a conservative hazard ratio of 1.2 for developing one of the primary endpoints, with significance of 0.05 and power of 0.8, we estimate that we would need we would need at least 945 patients with each diagnosis recorded and a comparator cohort of at least 15,742 controls. We consider this to be feasible, given the size of the database.

For project C, we calculate that we would need 2,873 cases with an exposure probability in the control group of 0.1 (or 5,376 cases for an exposure probability of 0.05) to estimate an OR of 1.2 with significance of 0.05 and power of 0.8, considering a matching of 1:5. We consider this to be feasible based on the number of adolescents documented with endometriosis in previous UK based population cohort studies.

Plan for missing data.

We anticipate missing data for ethnicity, body mass index, and smoking status. We will assume that these data are missing at random.

For peer review only

Missing values will be imputed using multiple imputation with chained equations using all outcome and exposure variables in the model. Five imputations will be carried out and Rubin's rules will be used to pool model estimates across the imputed datasets.

Sensitivity analysis

1. Complete vs imputed dataset

We will do a complete-case analysis as sensitivity analysis to check the missing at random (MAR) assumption. Results from both multiple imputation and complete case analyses will be reported.

2. Estimation of the rate ratio of endometriosis diagnosis in adolescence in those with and without documented coding of dysmenorrhoea

Case-control study C explores cases of endometriosis, with controls with and without dysmenorrhoea. We will also conduct a sensitivity analysis within the cohort coded with dysmenorrhoea with cases being those with dysmenorrhoea and adolescent endometriosis, and controls with dysmenorrhoea without diagnosed adolescent endometriosis, to identify other relevant patterns or variables that may affect or predict risk.

Ethics:

The project has been independently peer-reviewed and received ethics approval from the QResearch Scientific Board (reference OX46 under REC 18/EM/0400)[35.]

Dissemination:

Our findings will be reviewed with our PPI advisers, who will advise on dissemination and communication approaches. The findings of these studies will be published in peer reviewed academic journals and presented at conferences, including seeking cross-disciplinary avenues for research dissemination. We will work with collaborating advocacy organisations and patient groups to seek opportunities to share what we have learned and consider opportunities for how this can be utilised to improve care.

We will utilise the combined findings to generate an evidence-based infographic to support shared decision making about dysmenorrhoea in community care settings.

Finally, these projects will inform a subsequent qualitative study, exploring adolescent's experiences of menstrual pain. With their input, we will write accessible summaries about important aspects of painful periods which will be freely available on a widely used health experience website (Healthtalk.org).

Discussion:

This study explores the interface between a commonly experienced symptom in adolescence and the likelihood of subsequent diagnoses including endometriosis. By doing this, we seek to identify factors that will help clinicians and patients navigate their care journeys which will likely begin in primary care with presentation with as yet unexplained symptoms. These epidemiological studies will inform a subsequent qualitative study exploring young people's experiences of menstrual pain, including those who have (or have not) sought care, and who do (or do not) have a diagnosis of endometriosis.

The paucity of evidence in this field has led us to propose an initial comparison of cases (all adolescents coded with dysmenorrhoea) against controls without coded dysmenorrhea. Dysmenorrhea is common, affecting approximately 70-90% of the adolescent population. It is not known who, how, and when individuals present to primary care and we do not know how complete recording or coding is. Therefore it is likely that some of our control group will also experience dysmenorrhoea.

For young people deciding whether to access care, educators, families and front-line gate keeper clinicians, an important initial question is "how likely is it that any young person with period pain has an underlying condition?", "What can I advise about long-term outcomes?" and "What can we advise about treatments now? By including all adolescents who either did or did not present with dysmenorrhoea when we explore the likelihood of a diagnosis of

For peer review only

endometriosis, means that if there are systemic or structural reasons that affect attendance for care, then we may be able to consider these.

However, knowing how the risk of endometriosis can be stratified among adolescents who do present to primary care with dysmenorrhea is also important. Recognising this, we plan to undertake a sensitivity analysis contrasting rates of endometriosis in adolescents with documented dysmenorrhoea within study C, to see whether there are factors that help identify which adolescents with dysmenorrhea may be more likely to have endometriosis.

Understanding more about which adolescents with dysmenorrhoea to refer for further investigations is critical to improving care journeys. Knowing more about the impacts of treatment with hormonal medications in adolescence is also crucial to inform guidance for adolescents, their families, and health professionals who look after them.

The aims of this project align with health policy within the Women's Health Strategy which reinforces the need for awareness and resources to support menstrual wellbeing and care for those with endometriosis[36]

This approach will bridge and add to existing literature, which does not focus on the primary care interface crucial to accessing care in the UK health system.

A limitation of this project is its reliance on primary care coding, which may be incomplete or inaccurate. Data entry and recording in primary care is conducted to support clinical care and not predominantly to support research. However, existing understanding of endometriosis symptomatology in primary care is derived using comparable methods, although adolescents are not considered or reported separately[15-17]. Therefore, there is methodological justification for utilising this approach to consider adolescents as proposed in this study.

Limitations include the possibility of bias due to missing data, or from data misclassification, for example miscoding of symptoms or diagnoses. Adolescents with dysmenorrhoea may not have this documented in their primary care record, either because they have not attended for care, not reported this (or been heard), or not had this recorded as a Snomed code (for example if it is documented within consultation free text).

With menstrual symptoms, social acceptability bias may exist and may not be uniformly experienced across the population. The stratified analysis in project A may highlight areas for exploration in the aligned qualitative study. Recorder and recall bias are potentially relevant, whereby symptoms are better coded when a referral or treatment is planned, which will not apply to the comparator group.

Smoking and BMI are more likely to be recorded for young people prescribed hormonal treatment as these prescriptions represent a template driven opportunity to record this data. This data is more likely to be complete for young people prescribed hormonal treatment in primary care (and would not capture this data for those whose hormonal treatment was prescribed in other healthcare settings for example community sexual health clinics). Furthermore, the adolescents using sexual health clinics (rather than primary care) may systematically differ from each other (for example if they vary by location such as rural settings). Patterns of contraception use may also change over time, as demonstrated by the 'pill scare' of 1995 which caused a reduction in contraceptive pill use[37]. Menstruation and endometriosis do not link with any QOF codes, although long-acting contraception has done[38], which may act as a driver towards contraception being coded rather than pain or symptoms. Adolescence to adulthood can be a time of mobility, for example if adolescents leave home to travel elsewhere for work or study, which might limit the duration of follow up if people de-register from their GP when they move. Furthermore, these changes may not be uniformly distributed across social or demographic factors.

Finally, adolescent gynaecological units are not uniformly distributed across the country, and access to specialist adolescent services, tertiary or super-specialist care may influence referral rates and patterns of identification and referral[39]. If identified through stratification against region, this could offer a valuable finding.

We recognise that these represent a significant limitation of this approach. We will perform sensitivity analyses to compare robustness of results between complete-case and imputed analyses.

However, this project proposes a novel approach to develop understanding of a complex and important challenge when moving from symptoms of menstrual pain in adolescence to diagnosis of endometriosis, which is an under-researched and under-recognised health condition, which can have significant adverse impacts on women's health and wellbeing.

For peer review only - http://bmjopen.bmj.com/site/about/guidelines.xhtml

For peer review only

COI

KV has received research funding from Bayer Healthcare and honoraria for talks and consultancy fees from Bayer Healthcare, AbbVie and Eli Lilly. The remaining authors declare no relevant COI.

Funding:

This study was funded as part of Sharon Dixon's NIHR Doctoral research fellowship NIHR301787. There is no other relevant funding to declare. The views expressed are those of the author(s) and not necessarily those of the NIHR or the Department of Health and Social Care.

Author Contributions:

SD, KV and J H-C conceived and developed this proposed study. SD, J H-C, TR and MP developed the study design and statistical plan, including sample size calculation and protocol development. SD, J-H-C, KV and JB developed the data extraction plan and study code groups. SD, J H-C, MP, TR, and AS contributed to the proposed plan for data acquisition and curation. SD initially drafted this manuscript. All authors contributed to developing, refining and writing this manuscript.

Acknowledgments:

With thanks to Sue Ziebland, Lisa Hinton and Abigail McNiven for advice and guidance during the development of this project.

With enormous thanks to the study PPIE advisers, for their wisdom, encouragement and support.

References:

1. Parker, M.A., Sneddon, A.E. and Arbon, P., 2010. The menstrual disorder of teenagers (MDOT) study: determining typical menstrual patterns and menstrual disturbance in a large population-based study of Australian teenagers. BJOG: An International Journal of Obstetrics & Gynaecology, 117(2), pp.185-192
2. Suvitie, P.A., Hallamaa, M.K., Matomäki, J.M., Mäkinen, J.I. and Perheentupa, A.H., 2016. Prevalence of pain symptoms suggestive of endometriosis among Finnish adolescent girls (TEENMAPS study). Journal of pediatric and adolescent gynecology, 29(2), pp.97-103.
3. Vincenzo De Sanctis, M., Soliman, A., Bernasconi, S., Bianchin, L., Bona, G., Bozzola, M., Buzi, F., De, C., Sanctis, M.D., Tonini, G. and Rigon10, F., 2015. Primary dysmenorrhea in adolescents: prevalence, impact and recent knowledge. Pediatric Endocrinology Reviews (PER), 13(2), pp.465-73
4. Deb S, Raine-Fenning N. Dysmenorrhoea. Obstetrics, Gynaecology & Reproductive Medicine. 2008 Nov 1;18(11):294-9.
5. Smorgick N, Marsh CA, As-Sanie S, Smith YR, Quint EH. Prevalence of pain syndromes, mood conditions, and asthma in adolescents and young women with endometriosis. Journal of pediatric and adolescent gynecology. 2013 Jun 1;26(3):171-5
6. Vincent K, Warnaby C, Stagg CJ, Moore J, Kennedy S, Tracey I. Dysmenorrhoea is associated with central changes in otherwise healthy women. Pain. 2011 Sep 1;152(9):1966-75
7. Johnson, J., 1988. Level of knowledge among adolescent girls regarding effective treatment for dysmenorrhea. Journal of Adolescent Health Care, 9(5), pp.398
8. Armour M, Parry K, Al-Dabbas MA, Curry C, Holmes K, MacMillan F, Ferfolja T, Smith CA. Self-care strategies and sources of knowledge on menstruation in 12,526 young women with dysmenorrhea: A systematic review and meta-analysis. PLoS One. 2019 Jul 24;14(7):e0220103.
9. Houston, A.M., Abraham, A., Huang, Z. and D'Angelo, L.J., 2006. Knowledge, attitudes, and consequences of menstrual health in urban adolescent females. Journal of Pediatric and Adolescent Gynecology, 19(4), pp.271-275
10. De Sanctis V, Matalliotakis M, Soliman AT, Elsefdy H, Di Maio S, Fiscina B. A focus on the distinctions and current evidence of endometriosis in adolescents. Best Practice & Research Clinical Obstetrics & Gynaecology. 2018 Aug 1;51:138-50.

11. Pugsley, Z. and Ballard, K., 2007. Management of endometriosis in general practice: the pathway to diagnosis. Br J Gen Pract, 57(539), pp.470-476.
12. Johnston, J.L., Reid, H. and Hunter, D., 2015. Diagnosing endometriosis in primary care: clinical update. Br J Gen Pract, 65(631), pp.101-102#
13. Hirsch M, Dhillon-Smith R, Cutner A, Yap M, Creighton SM. The prevalence of endometriosis in adolescents with pelvic pain: A systematic review. Journal of Pediatric and Adolescent Gynecology. 2020 Jul 29. 24
14. Brosens, I., Gordts, S. and Benagiano, G., 2013. Endometriosis in adolescents is a hidden, progressive and severe disease that deserves attention, not just compassion. Human Reproduction, 28(8), pp.2026-2031
15. Ballard, K.D., Seaman, H.E., De Vries, C.S. and Wright, J.T., 2008. Can symptomatology help in the diagnosis of endometriosis? Findings from a national case–control study—Part 1. BJOG: An International Journal of Obstetrics & Gynaecology, 115(11), pp.1382-1391.
16. Cea Soriano, L., López-Garcia, E., Schulze-Rath, R. and Garcia Rodríguez, L.A., 2017. Incidence, treatment and recurrence of endometriosis in a UK-based population analysis using data from The Health Improvement Network and the Hospital Episode Statistics database. The European Journal of Contraception & Reproductive Health Care, 22(5), pp.334-343.
17. Burton, C., Iversen, L., Bhattacharya, S., Ayansina, D., Saraswat, L. and Sleeman, D., 2017. Pointers to earlier diagnosis of endometriosis: a nested case-control study using primary care electronic health records. Br J Gen Pract, 67(665), pp.e816-e823.
18. Ghai V, Jan H, Shakir F, Haines P, Kent A. Diagnostic delay for superficial and deep endometriosis in the United Kingdom. Journal of Obstetrics and Gynaecology. 2020 Jan 2;40(1):83-9.
19. Sieberg CB, Lunde CE, Borsook D. Endometriosis and pain in the adolescent-striking early to limit suffering: A narrative review. Neuroscience & Biobehavioral Reviews. 2020 Jan 1;108:866-76.
20. Janssen, E.B., Rijkers, A.C.M., Hoppenbrouwers, K., Meuleman, C. and d'Hooghe, T.M., 2013. Prevalence of endometriosis diagnosed by laparoscopy in adolescents with dysmenorrhea or chronic pelvic pain: a systematic review. Human reproduction update, 19(5), pp.570-582
21. Shim JY, Laufer MR. Adolescent endometriosis: an update. Journal of pediatric and adolescent gynecology. 2020 Apr 1;33(2):112-9.
22. Guideline of European Society of Human Reproduction and Embryology Endometriosis, https://www.eshre.eu/Guideline/Endometriosis, accessed 30.9.22
23. Chapron, C., Querleu, D., Bruhat, M.A., Madelenat, P., Fernandez, H., Pierre, F. and Dubuisson, J.B., 1998. Surgical complications of diagnostic and operative gynaecological laparoscopy: a series of 29,966 cases. Human reproduction (Oxford, England), 13(4), pp.867-872. 46.
24. Shah, D.K. and Missmer, S.A., 2011. Scientific investigation of endometriosis among adolescents. Journal of pediatric and adolescent gynecology, 24(5), pp.S18-S19
25. Clemenza S, Vannuccini S, Capezzuoli T, Meleca CI, Pampaloni F, Petraglia F. Is primary dysmenorrhea a precursor of future endometriosis development?. Gynecological Endocrinology. 2021 Apr 3;37(4):287-93.
26. Sarıdoğan, E., 2017. Adolescent endometriosis. European Journal of Obstetrics & Gynecology and Reproductive Biology, 209, pp.46-49
27. DiVasta, A.D., Vitonis, A.F., Laufer, M.R. and Missmer, S.A., 2018. Spectrum of symptoms in women diagnosed with endometriosis during adolescence vs adulthood. American journal of obstetrics and gynecology, 218(3), pp.324)
28. Yeung Jr P, Gupta S, Gieg S. Endometriosis in adolescents: a systematic review. Journal of Endometriosis and Pelvic Pain Disorders. 2017 Jan;9(1):17-29.
29. Randhawa AE, Tufte-Hewett AD, Weckesser AM, Jones GL, Hewett FG. Secondary School Girls' Experiences of Menstruation and Awareness of Endometriosis: A Cross-Sectional Study. Journal of pediatric and adolescent gynecology. 2021 Oct 1;34(5):643-8.
30. Williams CE, Creighton SM. Menstrual disorders in adolescents: review of current practice. Hormone research in Paediatrics. 2012;78(3):135-43.
31. Knox B, Ong YC, Bakar MA, Grover SR. A longitudinal study of adolescent dysmenorrhoea into adulthood. European journal of pediatrics. 2019 Sep 1;178(9):1325-32
32. Dixon S, McNiven A, Talbot A, Hinton L. Navigating possible endometriosis in primary care: a qualitative study of GP perspectives. British Journal of General Practice. 2021 Sep 1;71(710):e668-76.
33. QResearch, Home - QResearch, accessed 30.9.22

For peer review only - http://bmjopen.bmj.com/site/about/guidelines.xhtml

34. World Health Organisation, Adolescent health, https://www.who.int/health-topics/adolescent-health
35. What knowledge would support improving the management of teenagers with dysmenorrhoea in primary care? - QResearch, accessed 30.9.22
36. Women's health Strategy for England Policy paper, Women's Health Strategy for England - GOV.UK (www.gov.uk), accessed 30.9.22
37. Furedi A. The public health implications of the 1995 'pill scare'. Hum Reprod Update. 1999 Nov-Dec;5(6):621-6. doi: 10.1093/humupd/5.6.621. PMID: 10652971
38. Pasvol TJ, Macgregor EA, Rait G, Horsfall L. Time trends in contraceptive prescribing in UK primary care 2000–2018: a repeated cross-sectional study. BMJ sexual & reproductive health. 2022 Jul 1;48(3):193-8.
39. The British Society for Paediatric and Adolescent Gynaecology UK map of services, UK Map of Services – BritSPAG, accessed 30.9.22

For peer review only

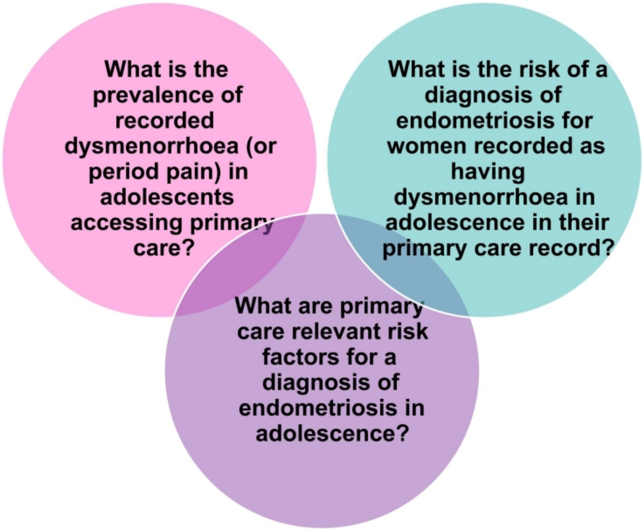

Figure 1: Schematic representation of the research aim and questions.

745x393mm (96 x 96 DPI)

STROBE Statement—checklist of items that should be included in reports of observational studies

	Item No	Recommendation
**Title and abstract**	1	(*a*) Indicate the study's design with a commonly used term in the title or the abstract.
		Page 1
		(*b*) Provide in the abstract an informative and balanced summary of what was done and what was found<br>This paper is a protocol, and therefore does not include findings, but a clear proposal for what we seek to explore and do within the study. Page 2.
**Introduction**		
Background/rationale	2	Explain the scientific background and rationale for the investigation being reported<br>Page 3
Objectives	3	State specific objectives, including any prespecified hypotheses<br>Page 4. (we have no pre-specified hypothesis, but set out our objectives).
**Methods**		
Study design	4	Present key elements of study design early in the paper<br>We introduce the method section with a clear description of study design, including a figure illustrating the key elements of the study design. Page 4. These are summarised within table 1, page 5.
Setting	5	Describe the setting, locations, and relevant dates, including periods of recruitment, exposure, follow-up, and data collection<br>Setting: UK General practice within the QResearch database, described page 5.<br>Study period summary for the project overall: Page 8
Participants	6	(*a*) *Cohort study*—Give the eligibility criteria, and the sources and methods of selection of participants. Describe methods of follow-up<br>This is referred to as project B and is described on page 8/9<br>*Case-control study*—Give the eligibility criteria, and the sources and methods of case ascertainment and control selection. Give the rationale for the choice of cases and controls<br>This is referred to as project C and is described on page 9/10<br>*Cross-sectional study*—Give the eligibility criteria, and the sources and methods of selection of participants<br>This is referred to as project A and is summarised on page 8
		(*b*) *Cohort study*—For matched studies, give matching criteria and number of exposed and unexposed<br>This is described in 'inclusion criterion' for project B, page 9<br>*Case-control study*—For matched studies, give matching criteria and the number of controls per case<br>This is described in 'inclusion criterion' for project C, page 10
Variables	7	Clearly define all outcomes, exposures, predictors, potential confounders, and effect modifiers. Give diagnostic criteria, if applicable<br>The variables included (including exposures, outcomes and potential confounders) are summarised in Table 2 (page 6-8).<br>Diagnostic criterion not applicable, as we are documenting/describing coding as documented by clinicians in a large database of routinely collected healthcare data.

For peer review only - http://bmjopen.bmj.com/site/about/guidelines.xhtml

Data sources/ measurement	8*	For each variable of interest, give sources of data and details of methods of assessment (measurement). Describe comparability of assessment methods if there is more than one group<br>This is detailed within table 2, page 6-8 and within the section titled data source and variables (page 6).
Bias	9	Describe any efforts to address potential sources of bias<br>We have described our plans for missing data and our proposed sensitivity analysis. Page 11.<br>We also describe the biases we anticipate (page 11-13).
Study size	10	Explain how the study size was arrived at<br>This is detailed on page 10/11
Quantitative variables	11	Explain how quantitative variables were handled in the analyses. If applicable, describe which groupings were chosen and why<br>N/A this is a protocol paper
Statistical methods	12	(*a*) Describe all statistical methods, including those used to control for confounding
		(*b*) Describe any methods used to examine subgroups and interactions
		(*c*) Explain how missing data were addressed
		(*d*) *Cohort study*—If applicable, explain how loss to follow-up was addressed<br>*Case-control study*—If applicable, explain how matching of cases and controls was addressed<br>*Cross-sectional study*—If applicable, describe analytical methods taking account of sampling strategy
		(*e*) Describe any sensitivity analyses<br>We describe our protocol plan to address the statistical analysis, for all of the points above. (page 8-10)

Continued on next page

For peer review only - http://bmjopen.bmj.com/site/about/guidelines.xhtml

Results **N/A: protocol paper**

Participants	13*	(a) Report numbers of individuals at each stage of study—eg numbers potentially eligible, examined for eligibility, confirmed eligible, included in the study, completing follow-up, and analysed
		(b) Give reasons for non-participation at each stage
		(c) Consider use of a flow diagram
Descriptive data	14*	(a) Give characteristics of study participants (eg demographic, clinical, social) and information on exposures and potential confounders
		(b) Indicate number of participants with missing data for each variable of interest
		(c) *Cohort study*—Summarise follow-up time (eg, average and total amount)
Outcome data	15*	*Cohort study*—Report numbers of outcome events or summary measures over time
		Case-control study—Report numbers in each exposure category, or summary measures of exposure
		Cross-sectional study—Report numbers of outcome events or summary measures
Main results	16	(*a*) Give unadjusted estimates and, if applicable, confounder-adjusted estimates and their precision (eg, 95% confidence interval). Make clear which confounders were adjusted for and why they were included
		(*b*) Report category boundaries when continuous variables were categorized
		(*c*) If relevant, consider translating estimates of relative risk into absolute risk for a meaningful time period
Other analyses	17	Report other analyses done—eg analyses of subgroups and interactions, and sensitivity analyses

Discussion **N/A: protocol paper**

Key results	18	Summarise key results with reference to study objectives
Limitations	19	Discuss limitations of the study, taking into account sources of potential bias or imprecision. Discuss both direction and magnitude of any potential bias
Interpretation	20	Give a cautious overall interpretation of results considering objectives, limitations, multiplicity of analyses, results from similar studies, and other relevant evidence
Generalisability	21	Discuss the generalisability (external validity) of the study results

Other information

Funding	22	Give the source of funding and the role of the funders for the present study and, if applicable, for the original study on which the present article is based<br>This is reported on page 14 and the cover sheet (page 1).

*Give information separately for cases and controls in case-control studies and, if applicable, for exposed and unexposed groups in cohort and cross-sectional studies.

Note: An Explanation and Elaboration article discusses each checklist item and gives methodological background and published examples of transparent reporting. The STROBE checklist is best used in conjunction with this article (freely available on the Web sites of PLoS Medicine at http://www.plosmedicine.org/, Annals of Internal Medicine at http://www.annals.org/, and Epidemiology at http://www.epidem.com/). Information on the STROBE Initiative is available at www.strobe-statement.org.

For peer review only

For peer review only - http://bmjopen.bmj.com/site/about/guidelines.xhtml
