# Supplementary material for: Exploring the interface between adolescent dysmenorrhoea and endometriosis: a protocol for a cohort and nested case–control study within the QResearch Database
Source: BMJ Open. 2023 Feb 14;13(2):e069984. doi: 10.1136/bmjopen-2022-069984 (PMC9930556; doi:10.1136/bmjopen-2022-069984)
Supplement: Reviewer comments [file bmjopen-2022-069984.reviewer_comments.pdf]

PEER REVIEW HISTORY

BMJ Open publishes all reviews undertaken for accepted manuscripts. Reviewers are asked to complete a checklist review form (**http://bmjopen.bmj.com/site/about/resources/checklist.pdf**) and are provided with free text boxes to elaborate on their assessment. These free text comments are reproduced below.

ARTICLE DETAILS

**TITLE (PROVISIONAL)**	Exploring the interface between adolescent dysmenorrhoea and endometriosis: A protocol for a cohort and nested case-control study within the QResearch Database
**AUTHORS**	Dixon, Sharon; Ranger, Tom; Burchardt, Judith; Patone, Martina; Snelling, Andrew; Vincent, Katy; Hippisley-Cox, Julia

This article was not externally reviewed at BMJ Open. Protocols that have been independently assessed prior to submission to BMJ Open are usually fast-tracked to publication on the grounds that further substantial changes will not be possible. This independent assessment will usually be external, independent review for both a substantial grant award from a non-commercial or government funder and ethics approval.
